# Supplementary material for: Combining Stage Specificity and Metabolomic Profiling to Advance Antimalarial Drug Discovery
Source: Cell Chem Biol. 2020 Feb 20;27(2):158–171.e3. doi: 10.1016/j.chembiol.2019.11.009 (PMC7031696; doi:10.1016/j.chembiol.2019.11.009)
Supplement: Document S1. Figures S1–S4 and Tables S1–S3 [file mmc1.pdf]

Cell Chemical Biology, Volume *27*

Supplemental Information

Combining Stage Specificity and Metabolomic Profiling to Advance Antimalarial Drug Discovery

James M. Murithi, Edward S. Owen, Eva S. Istvan, Marcus C.S. Lee, Sabine Ottilie, Kelly Chibale, Daniel E. Goldberg, Elizabeth A. Winzeler, Manuel Llinás, David A. Fidock, and Manu Vanaerschot

Table S1 (related to Figures 1–6). Asexual blood stage-specific IC_{50}^{8h} data in nM for the tested antimalarials.

Compound	Early rings			Late rings			Early trophozoites			Late trophozoites			Schizonts			Overall (72hr assay)			Peak Activity	Ratio lowest $IC_{50}^{8hr}/IC_{50}^{72hr}$
	Mean IC_{50}	SEM	n	Mean IC_{50}	SEM	n	Mean IC_{50}	SEM	n	Mean IC_{50}	SEM	n	Mean IC_{50}	SEM	n	Mean IC_{50}	SEM	n		
Dihydroartemisinin	1.5	0.1	3	2.4	0.1	3	2.4	0.4	3	3.9	0.2	3	11.6	1.3	3	1.0	0.1	4	Rings + Trophs	1.5
Chloroquine	17.8	5.5	4	18.6	2.5	3	17.4	3.5	3	30.1	6.3	3	60.3	13.4	3	5.9	0.8	3	Rings + Trophs	2.9
Mefloquine	258	106	3	147	74	3	28.8	7.0	3	29.5	6.9	3	138	39	3	9.8	1.0	3	Trophs	3.0
Lumefantrine	15.4	4.0	3	15.8	3.5	3	19.54	1.6	3	22.3	2.0	3	81.0	32.5	3	1.3	0.0	3	Rings + Trophs	11.6
Piperaquine	26.2	1.1	3	46.3	13.1	3	27.4	3.3	3	32.9	1.1	3	67.0	9.6	3	15.4	2.3	4	Rings + Trophs	1.7
Ferroquine	4.9	0.3	3	10.9	1.7	3	23.2	2.4	3	36.8	3.3	3	49.3	6.2	3	3.5	0.4	4	Rings	1.4
Methylene blue	3.2	0.3	3	13.3	2.7	3	5.9	0.4	3	8.3	2.0	3	64.8	17.8	3	1.8	0.1	4	Rings + Trophs	1.8
KAI407 - shift 1	-	-	-	-	-	-	-	-	-	161	44.0	3	-	-	-	-	-	-	-	3.9
KAI407 - shift 2	16263	904	2	14021	923	3	5139	1727	3	13386	354	2	46.2	0.6	3	11.8	1.0	3	Schizonts	-
AN3661	> 1.25 µM	-	3	> 1.25 µM	-	3	> 1.25 µM	-	3	138	39	3	400	34	3	35.6	3.6	3	Late Trophs	3.9
KAE609	> 1.25 µM	-	3	> 1.25 µM	-	3	3.2	0.6	3	1.1	0.2	3	1.2	0.1	3	0.73	0.05	4	Trophs + Schizonts	1.5
WLL-vs	23.7	2.6	3	29.4	4.4	3	57.6	3.1	3	47.1	3.1	3	50.1	5.7	3	7.2	0.8	3	Rings	3.3
DSM265 - shift 1	-	-	-	-	-	-	10.2	2.2	3	7.7	0.9	3	19.2	2.3	3	3.8	0.1	3	Late Trophs	2.0
DSM265 - shift 2	4821	1605	3	1493	308	3	1481	240	3	-	-	-	> 25 µM	-	-	-	-	-	-	-
Atovaquone - shift 1	-	-	-	-	-	-	0.68	0.06	3	3.7	0.2	3	7.4	1.3	3	1.1	0.1	3	Late Trophs	3.3
Atovaquone - shift 2	553	228	3	324	45	3	619	132	3	-	-	3	> 25 µM	-	3	-	-	-	-	-
Fosmidomycin	> 10 µM	-	3	> 10 µM	-	3	1983	665	3	1993	117	3	> 10 µM	-	3	405	8	3	Trophs	4.9
GNF-Pf-5660	119	30	3	238	57	3	108	6	3	79.3	10.8	3	290	50	3	4.4	0.2	3	Rings + Trophs	18.0
MMV665794	369	101	3	590	177	3	210	20	3	165	10	3	203	8	3	91.3	5.2	4	Trophs + Schizonts	1.8
Naphtoquine	4.2	1.8	3	8.1	0.4	3	19.5	2.9	3	29.2	3.1	3	42.8	8.4	3	3.2	0.4	4	Rings	1.3
MMV000442	> 8 µM	-	3	> 8 µM	-	3	176	11	3	197	22	3	503	48	3	37.2	3.1	3	Trophs	4.7
MMV675939	1170	277	3	268	84	3	62.9	29.9	3	146	54	3	1647	45	3	30.8	4.4	5	Trophs	2.0
MMV085071	893	78	3	492	122	3	61.1	0.1	3	81.4	2.8	3	254	47	3	105	11	3	Trophs	0.6
MMV668311	140	39	3	97.7	4.3	3	68.9	11.9	3	122	26	3	382	79	3	63.5	1.7	3	Rings + Trophs	1.1
MMV020746	> 25 µM	-	2	> 25 µM	-	2	12587	1681	2	> 25 µM	-	2	> 25 µM	-	2	56.8	6.6	4	All stages > 10 µM	221.5
MMV667491	2264	349	3	1270	92	3	521	28	3	731	50	3	842	108	3	243	35	4	Trophs + Schizonts	2.1
MMV006455	> 12.5 µM	-	3	> 12.5 µM	-	3	441	10	3	450	46	3	933	142	3	439	46	4	Trophs	1.0
MMV022478	7628	202	2	4870	353	2	2132	252	2	2529	353	2	3370	269	2	11.1	0.8	3	Trophs + Schizonts	192.4
MMV007181	> 25 µM	-	3	> 25 µM	-	3	253	27	3	274	16	3	570	107	3	84.9	8.9	3	Trophs	3.0
MMV665971	> 25 µM	-	3	> 25 µM	-	3	496	51	3	485	26	3	523	29	3	185	9	3	Trophs + Schizonts	2.6
MMV665939	17011	2420	3	22853	5330	3	13476	4752	3	39017	13066	3	54384	14265	3	478	49	4	All stages > 10 µM	28.2
MMV019017	8418	1862	2	8663	2298	3	1338	133	3	1708	116	3	3060	229	3	306	12	3	Trophs + Schizonts	4.4
MMV000248	n.d.	n.d.	0	6744	-	1	411	-	1	376	-	1	754	-	1	103	14	4	Trophs	3.7
MMV021735	> 25 µM	-	3	> 25 µM	-	3	5422	1392	3	234	12	3	5281	622	3	232	11	4	Late Trophs	1.0
MMV022224	838	123	3	845	152	3	542	39	3	752	58	3	2486	472	3	199	31	5	Rings + Trophs	2.7
MMV027496	2760	529	3	878	130	3	168	26	3	199	9	3	4339	545	3	89.7	7.9	3	Trophs	1.9
MMV019555	755	159	3	788	221	3	81.4	1.6	3	110	20	3	349	66	3	33.3	1.8	3	Trophs	2.4
MMV030666	10264	3053	3	11440	861	3	5124	506	3	858	29	3	4191	307	3	462	18	4	Late Trophs	1.9
MMV000787	5737	1215	3	7870	731	3	3312	629	3	1354	57	3	5912	617	3	1960	83	3	Late Trophs	0.7

Shift 1 and shift 2 indicate the two half-maximal inhibitory concentrations for biphasic dose response curves. SEM: standard error of the mean; n: number of biological repeats; - : no data; IC_{50}^{8h}: IC_{50} based on 8-h exposure; IC_{50}^{72h}: IC_{50} based on 72-h exposure.

Table S2 (related to Figures 1–6). SMILES and suspected mode of action (if known) of the tested antimalarials.

Compound	SMILES	Suspected mode of action	Origin candidate antimalarials	Alternative name
Dihydroartemisinin (DHA)	C[C@@H]1CC[C@H]2[C@H]([C@H](O[C@H]3[C@@]24[C@H]1CCC(O3)(OO4)C)O)C	-	-	-
Chloroquine (CQ)	CCN(CC)CCCC(C)NC1=C2C=CC(=CC2=NC=C1)Cl	β-hematin[a]	-	-
Mefloquine (MFQ)	c1cc2c(cc(nc2c(c1)C(F)(F)F)C(F)(F)F)[C@@H]([C@H]3CCCCN3)O	-	-	-
Lumefantrine (LMF)	CCCCN(CCCC)CC(C1=C2C3=C(C=C(C=C3)Cl)C(=CC4=CC=C(C=C4)Cl)C2=CC(=C1)Cl)O	-	-	-
Piperaquine (PPQ)	c1cc2c(ccnc2cc1Cl)N3CCN(CC3)CCCN4CCN(CC4)c5ccnc6c5ccc(c6)Cl	β-hematin[b]	-	-
Ferroquine (FQ)	CN(C)CC1=C(C=[C-]C1)CNC2=C3C=CC(=CC3=NC=C2)Cl.C1C=CC=[C-]1.[Fe+2]	β-hematin[c,d]	-	-
Methylene blue (MB)	CN(C)C1=CC2=C(C=C1)N=C3C=CC(=[N+](C)C)C=C3S2.[Cl-]	β-hematin[e]	-	-
KAI407	O=C(N(C)C1=CC=C(C=C1)C#N)C2=CN3C(C=N2)=NC=C3C4=CC=C(C=C4)C(F)(F)F	PI4K[f]	-	-
AN3661	OB1C2=C(CCC(O)=O)C=CC=C2CO1	CPSF[g]	-	-
KAE609	ClC1=C(F)C=C2C(NC3=C2C[C@H](C)N[C@@]34C(CC5=C4C=C(Cl)C=C5)=O)=C1	ATP4[h]	-	-
WLL-vs	CC(C)C[C@@H](/C=C/S(=O)(=O)C)NC(=O)[C@H](CC(C)C)NC(=O)[C@H](CC1=CNC2=CC=CC=C21)NC(=O)CN3CCOCC3	Proteasome[i,j]	-	-
DSM265	FS(F)(F)(F)(C1=CC=C(NC2=CC(C)=NC3=NC(C(F)(F)C)=NN23)C=C1)F	DHODH[k]	-	-
Atovaquone (ATQ)	O=C1C([C@@H]2CC[C@@H](C3=CC=C(Cl)C=C3)CC2)=C(O)C(C4=CC=CC=C41)=O	CYTB[l]	-	-
Fosmidomycin	C(CN(C=O)O)CP(=O)(O)O	DXR[m]	-	-
GNF-Pf-5660	CCOC(=O)C1=C(C)NC2=C(C1C3=CC=CC=C3Cl)C(=O)CC(C2)C4=CC=C(OC)C(OC)=C4	*[n]	-	-
Naphthoquine (NQ)	CC(C)(C)NCc1cc(c2c(c1O)CCCC2)Nc3ccnc4c3ccc(c4)Cl	-	-	MMV000017
MMV665794	FC(F)(F)C1=CC(NC2=C(NC3=CC(=CC=C3)C(F)(F)F)N=C3C=CC=CC3=N2)=CC=C1	-	MMV Malaria Box	-
MMV000442	CC(C)(C)c1ccc2OCN(Cc3ccc(Cl)cc3)Cc2c1	-	MMV Malaria Box	-
MMV675939	FC(F)(F)C1=CC=C(NC2=CC(NC(C3=CC(C(F)(F)F)=CC=C3)=N4)=C4C=N2)N=C1	-	Literature[o]	-
MMV085071	COc1cncc(c1)-c1cncc(n1)N1CCN(CC1)c1ccncc1	-	MMV Pathogen Box	-
MMV668311	CNc1nc(NCCCN(C)C)c2sc(cc2n1)c3cccc(c3)C(F)(F)F	-	Literature[p]	-
MMV020746	Cc1ccc(Oc2ncccc2C(=O)Nc2cccc3cccnc23)c(C)c1	-	Literature[p]	TCMDC-125499
MMV667491	CN(C)CCCn1cnc2c(c1=N)C(c3ccc4ccccc4c3O2)c5ccc(cc5)OC	-	MMV Malaria Box	-
MMV006455	CCCN(CCC)CC(O)COC1=C(C=CC=C1)C(=O)NC1=CC=CC=C1	-	MMV Malaria Box	-
MMV022478	Clc1cccc(c1)-c1cnn2ccc(nc12)C(=O)Nc1ccc(cc1)N1CCNCC1.OC(C(F)(F)F)=O	-	MMV Pathogen Box	-
MMV007181	CC1=C2C=CC(O)=CC2=NC(NC2=CC=C(OCC3=CC=CC=C3)C=C2)=C1	-	MMV Malaria Box	-
MMV665971	CCOC(=O)C1=C(C)N=c2s\c(=C/c3cc(Cl)ccc3O)c(=O)n2C1c1ccc(OC)cc1	-	MMV Malaria Box	-
MMV665939	FC1=CC=C(C=C1)C(=O)NC1=C(SC=C1)C(=O)NC1CCCCC1	-	MMV Malaria Box	-
MMV019017	COCCNCC(O)CN1C2=CC=C(Cl)C=C2C2=C1C=CC(Cl)=C2	-	MMV Malaria Box	-
MMV000248	Cl.CCN(CC)CCn1c2ccccc2n(CC(O)c2ccc(Cl)c(Cl)c2)c1=N	-	MMV Malaria Box	-
MMV021735	CCCCCCCN(CC1=CC=C(OC(C)(C)C(=O)OCC)C=C1)C(=O)NC1=CC=C(Cl)C=C1OCC	-	Literature[p]	TCMDC-131919
MMV022224	[O-]C(=O)C(F)(F)F.CN(C)CC1=CC=C(C=C1)C1=CC2=C(N1)N=CC=C2C1=CC=C(CN(C)C)C=C1	-	Literature[p]	TCMDC-132409
MMV027496	COC1=C(OCCN(C)C)C=CC(=C1)C1=NC(=C(N1)C1=CC=CC=C1)C1=CC=CC=C1	-	Literature[p]	TCMDC-137716
MMV019555	Cl.C(CCCNc1c2CCCCc2nc2ccccc12)CCNc1c2CCCCc2nc2ccccc12	-	MMV Malaria Box	TCMDC-124183
MMV030666	CC(C)(C)OC(=O)N1CCN(CC1)C1=CC=CC=C1NC(=O)C1=C(OC2=CC=C(F)C=C2)C(=CC=C1)C(F)(F)F	-	Literature[p]	TCMDC-140951
MMV000787	CCCOCC1=C2C=CC=NC2=C(O)C(CN2CCN(CC2)C2=CC(Cl)=CC=C2)=C1	-	MMV Malaria Box	-

PI4K: Phosphatidylinositol-4-OH kinase, CPSF: Cleavage and polyadenylation specificity factor, ATP4: P-type cation translocating ATPase, DHODH: Dihydroorotate dehydrogenase, CYTB: Cytochrome B, DXR: 1-deoxy-D-xylulose-5-phosphate reductoisomerase; SMILES: simplified molecular line entry system.

[a] Slater, A.F., and Cerami, A. (1992). Inhibition by chloroquine of a novel haem polymerase enzyme activity in malaria trophozoites. Nature *355*, 167-169.

[b] Raynes, K., Foley, M., Tilley, L., and Deady, L.W. (1996). Novel bisquinoline antimalarials. Synthesis, antimalarial activity, and inhibition of haem polymerisation. Biochemical Pharmacology *52*, 551-559.

[c] Biot, C., Taramelli, D., Forfar-Bares, I., Maciejewski, L.A., Boyce, M., Nowogrocki, G., Brocard, J.S., Basilico, N., Olliaro, P., and Egan, T.J. (2005). Insights into the mechanism of action of ferroquine. Relationship between physicochemical properties and antiplasmodial activity. Molecular Pharmaceutics *2*, 185-193.

[d] Dubar, F., Egan, T.J., Pradines, B., Kuter, D., Ncokazi, K.K., Forge, D., Paul, J.F., Pierrot, C., Kalamou, H., Khalife, J., et al. (2011). The antimalarial ferroquine: role of the metal and intramolecular hydrogen bond in activity and resistance. ACS Chemical Biology *6*, 275-287.

[e] Atamna, H., Krugliak, M., Shalmiev, G., Deharo, E., Pescarmona, G., and Ginsburg, H. (1996). Mode of antimalarial effect of methylene blue and some of its analogues on Plasmodium falciparum in culture and their inhibition of P. vinckei petteri and P. yoelii nigeriensis in vivo. Biochemical Pharmacology *51*, 693-700.

[f] McNamara, C.W., Lee, M.C., Lim, C.S., Lim, S.H., Roland, J., Simon, O., Yeung, B.K., Chatterjee, A.K., McCormack, S.L., Manary, M.J., et al. (2013). Targeting Plasmodium PI(4)K to eliminate malaria. Nature *504*, 248-253.

[g] Sonoiki, E., Ng, C.L., Lee, M.C., Guo, D., Zhang, Y.K., Zhou, Y., Alley, M.R., Ahyong, V., Sanz, L.M., Lafuente-Monasterio, M.J., et al. (2017). A potent antimalarial benzoxaborole targets a Plasmodium falciparum cleavage and polyadenylation specificity factor homologue. Nature Communications *8*, 14574.

[h] Spillman, N.J., Allen, R.J., McNamara, C.W., Yeung, B.K., Winzeler, E.A., Diagana, T.T., and Kirk, K. (2013). Na(+) regulation in the malaria parasite Plasmodium falciparum involves the cation ATPase PfATP4 and is a target of the spiroindolone antimalarials. Cell Host Microbe *13*, 227-237.

[i] Stokes, B.H., Yoo, E., Murithi, J.M., Luth, M.R., Afanasyev, P., da Fonseca, P.C.A., Winzeler, E.A., Ng, C.L., Bogyo, M., and Fidock, D.A. (2019). Covalent Plasmodium falciparum-selective proteasome inhibitors exhibit a low propensity for generating resistance in vitro and synergize with multiple antimalarial agents. PLoS Pathog. 15, e1007722.

[j] Li, H., O'Donoghue, A.J., van der Linden, W.A., Xie, S.C., Yoo, E., Foe, I.T., Tilley, L., Craik, C.S., da Fonseca, P.C., and Bogyo, M. (2016). Structure- and function-based design of Plasmodium-selective proteasome inhibitors. Nature *530*, 233-236.

[k] Coteron, J.M., Marco, M., Esquivias, J., Deng, X., White, K.L., White, J., Koltun, M., El Mazouni, F., Kokkonda, S., Katneni, K., et al. (2011). Structure-guided lead optimization of triazolopyrimidine-ring substituents identifies potent Plasmodium falciparum dihydroorotate dehydrogenase inhibitors with clinical candidate potential. Journal of Medicinal Chemistry *54*, 5540-5561.

[l] Fry, M., and Pudney, M. (1992). Site of action of the antimalarial hydroxynaphthoquinone, 2-[trans-4-(4'-chlorophenyl) cyclohexyl]-3-hydroxy-1,4-naphthoquinone (566C80). Biochemical Pharmacology *43*, 1545-1553.

[m] Armstrong, C.M., Meyers, D.J., Imlay, L.S., Freel Meyers, C., and Odom, A.R. (2015). Resistance to the antimicrobial agent fosmidomycin and an FR900098 prodrug through mutations in the deoxyxylulose phosphate reductoisomerase gene (dxr). Antimicrobial Agents and Chemotherapy *59*, 5511-5519.

[n] Vanaerschot, M., Lucantoni, L., Li, T., Combrinck, J.M., Ruecker, A., Kumar, T.R.S., Rubiano, K., Ferreira, P.E., Siciliano, G., Gulati, S., et al. (2017). Hexahydroquinolines are antimalarial candidates with potent blood-stage and transmission-blocking activity. Nature Microbiology *2*, 1403-1414.

[o] Nchinda, A.T., Le Manach, C., Paquet, T., Gonzalez Cabrera, D., Wicht, K.J., Brunschwig C., Njoroge, M., Abay, E., Taylor, D., Lawrence, N., et al. (2018). Identification of fast-acting 2,6-disubstituted imidazopyridines that are efficacious in the in vivo humanized Plasmodium falciparum NODscidIL2Rγ^{null} mouse model of Malaria. Journal of Medicineal Chemistry *61*, 4213-4227.

[p] Corey, V.C., Lukens, A.K., Istvan, E.S., Lee, M.C.S., Franco, V., Magistrado, P., Coburn-Flynn, O., Sakata-Kato, T., Gnadig, N., et al. (2016). A broad analysis of resistance development in the malaria parasite. Nature Communications *15*, 11901.

Table S3 (related to Figure 4). Assessment of compound solubility by UV/vis spectroscopy.

Compound	Wavelength						Ratio fil./std.	Solubility
	280	300	320	340	360	800		
	Absorbance (AU)							
**DHA fil.**	2.351	0.462	0.250	0.179	0.124	0.041	1.02	≥ 500 µM
**DHA std.**	2.314	0.456	0.244	0.173	0.120	0.042		
**Chloroquine fil.**	2.637	1.170	2.155	2.075	0.203	0.042	0.91	≥ 100 µM and ≤ 500 µM
**Chloroquine std.**	2.747	1.331	2.562	2.178	0.204	0.041		
**Piperaquine fil.**	2.322	0.473	0.269	0.189	0.129	0.041	0.69	≥ 100 µM and ≤ 500 µM
**Piperaquine std.**	2.694	0.822	0.646	0.555	0.462	0.304		
**MMV007181 fil.**	2.346	0.470	0.259	0.194	0.139	0.042	0.69	≥ 100 µM and ≤ 500 µM
**MMV007181 std.**	2.677	0.754	0.534	0.551	0.508	0.131		
**MMV000442 fil.**	2.517	0.606	0.380	0.310	0.248	0.086	0.74	≥ 100 µM and ≤ 500 µM
**MMV000442 std.**	2.754	0.898	0.711	0.660	0.619	0.274		
**MMV006455 fil.**	2.810	0.904	0.336	0.186	0.130	0.041	1.00	≥ 500 µM
**MMV006455 std.**	2.826	0.928	0.329	0.175	0.120	0.041		

Fil.: filtered; std: standard.

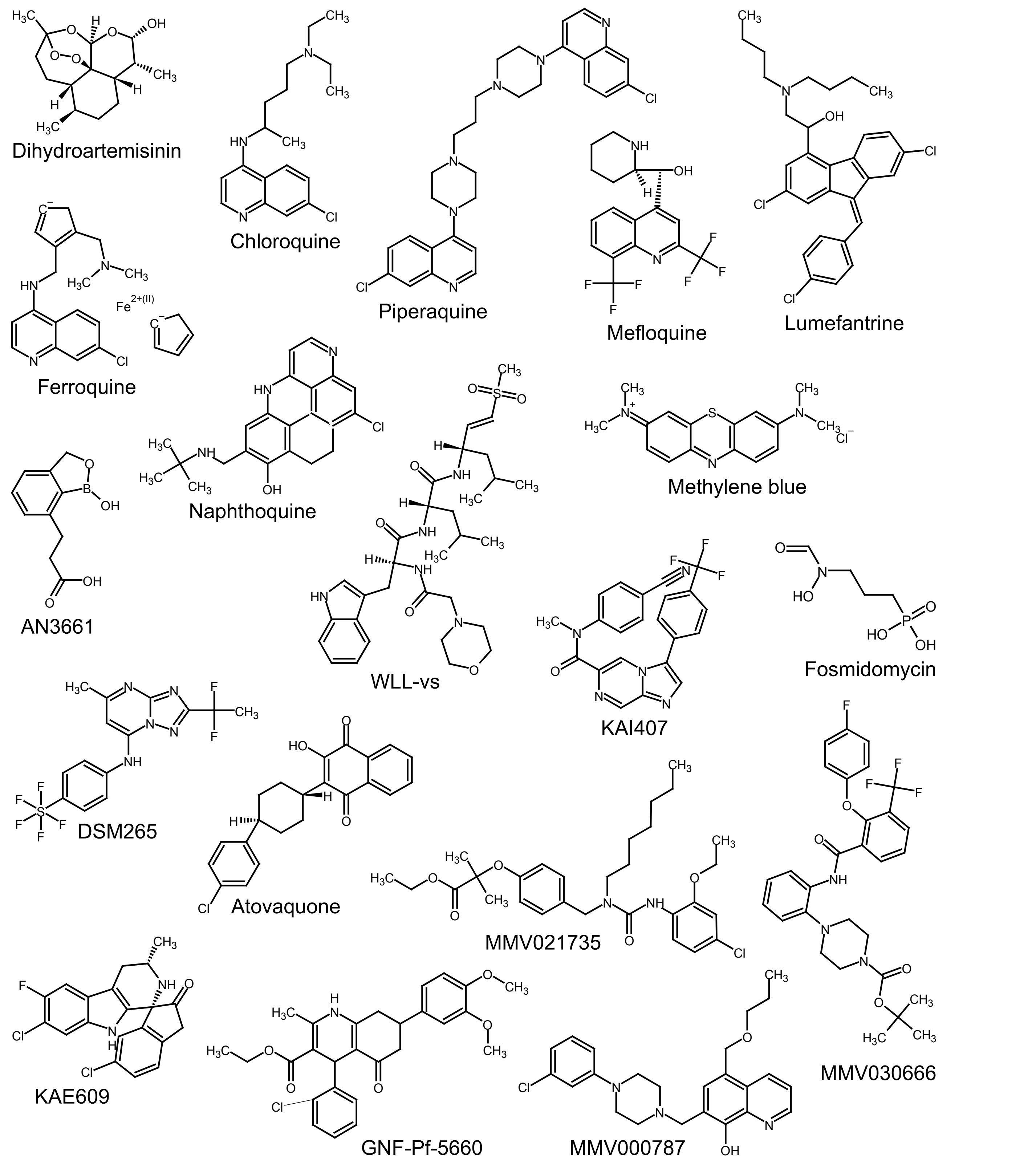

Figure S1 (related to Figures 1–6). Structures of the tested antimalarials, part 1. References on mode of action can be found in Table S2.

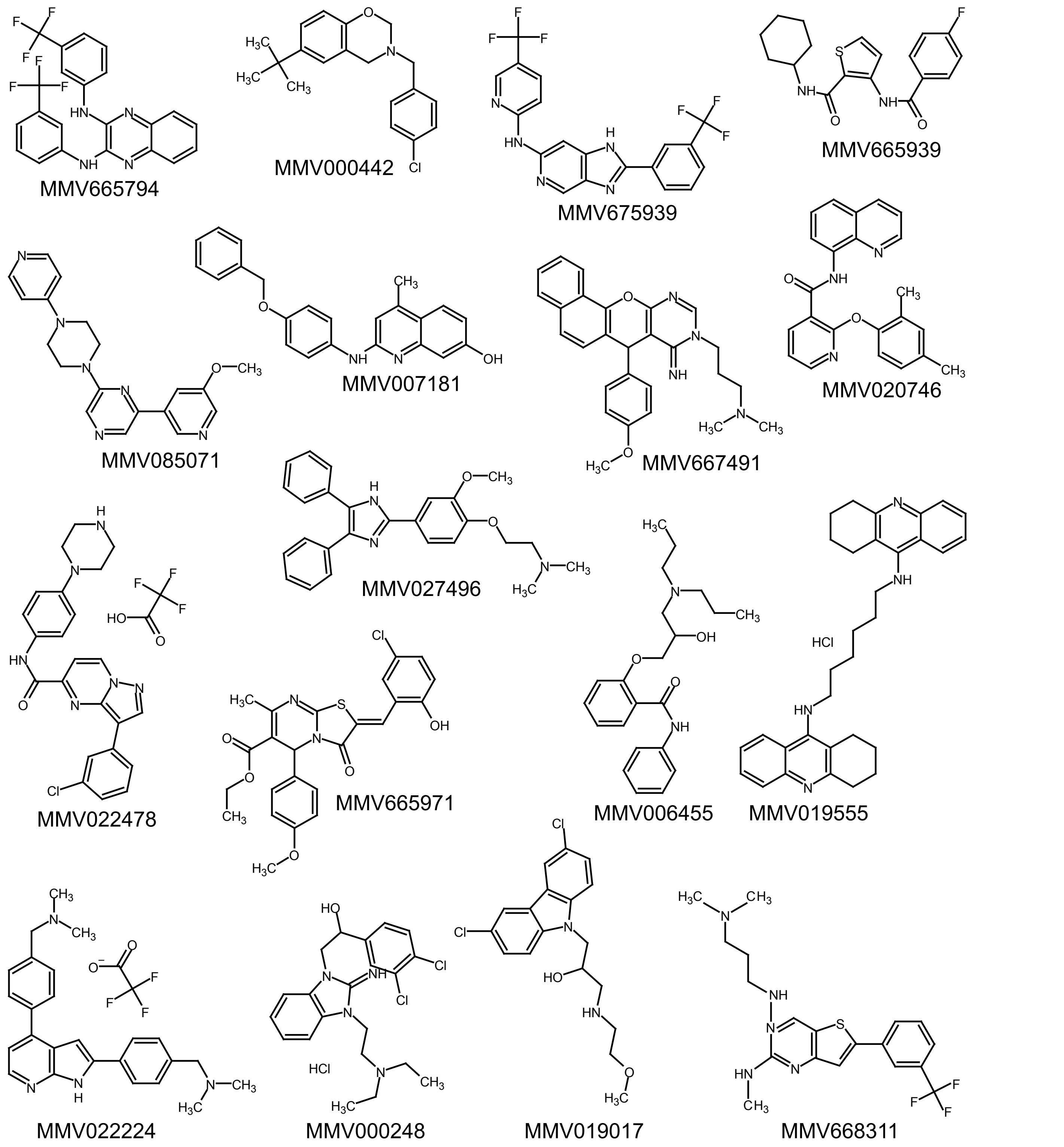

Figure S2 (related to Figures 1–6). Structures of the tested antimalarials, part 2. References on mode of action can be found in Table S2.

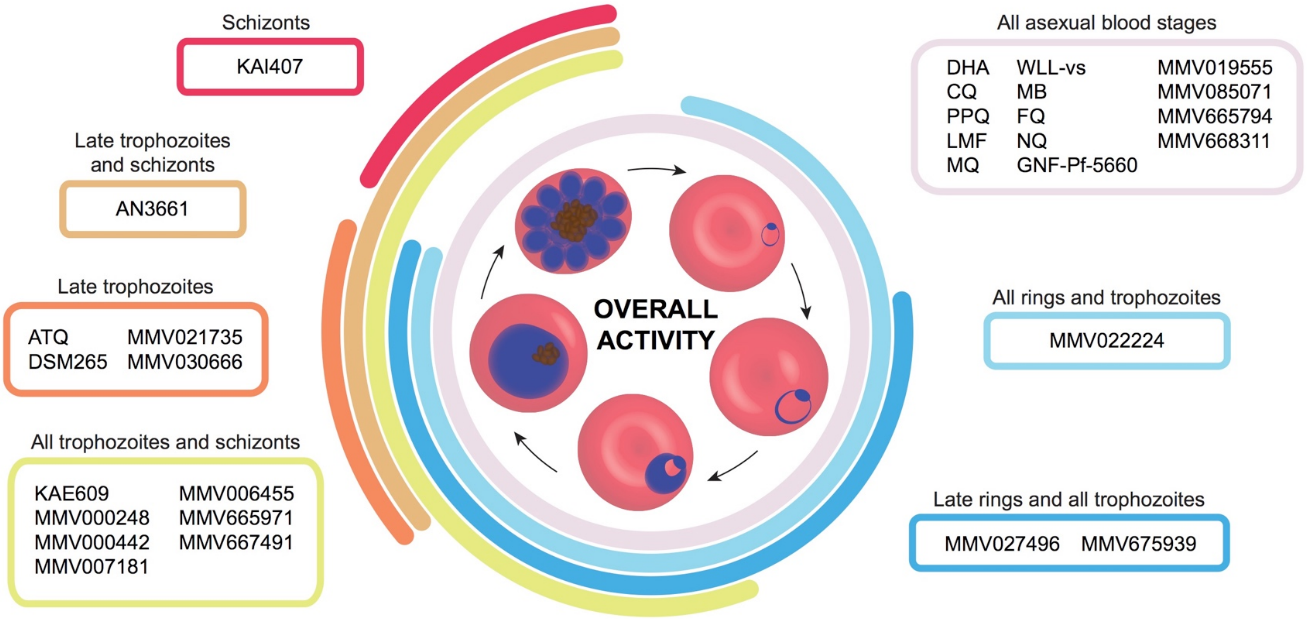

Figure S3 (related to Figure 6). Overall activity profile of compounds. Overall activity is defined as the stages with IC_{50}^{8h} values < 1 µM. Fosmidomycin, MMV000787, MMV019017, MMV020746, MMV022478 and MMV665939 are not depicted, as all stages showed IC_{50}^{8hr} values > 1 µM. MMV006455, MMV000442, MMV007181 and MMV665971 are omitted from panel A due to incomplete killing at individual stages. DHA: dihydroartemisinin; CQ: chloroquine; PPQ: piperaquine; LMF: lumefantrine; MQ: mefloquine; MB: methylene blue; FQ: ferroquine; NQ: naphthoquine; ATQ: atovaquone.

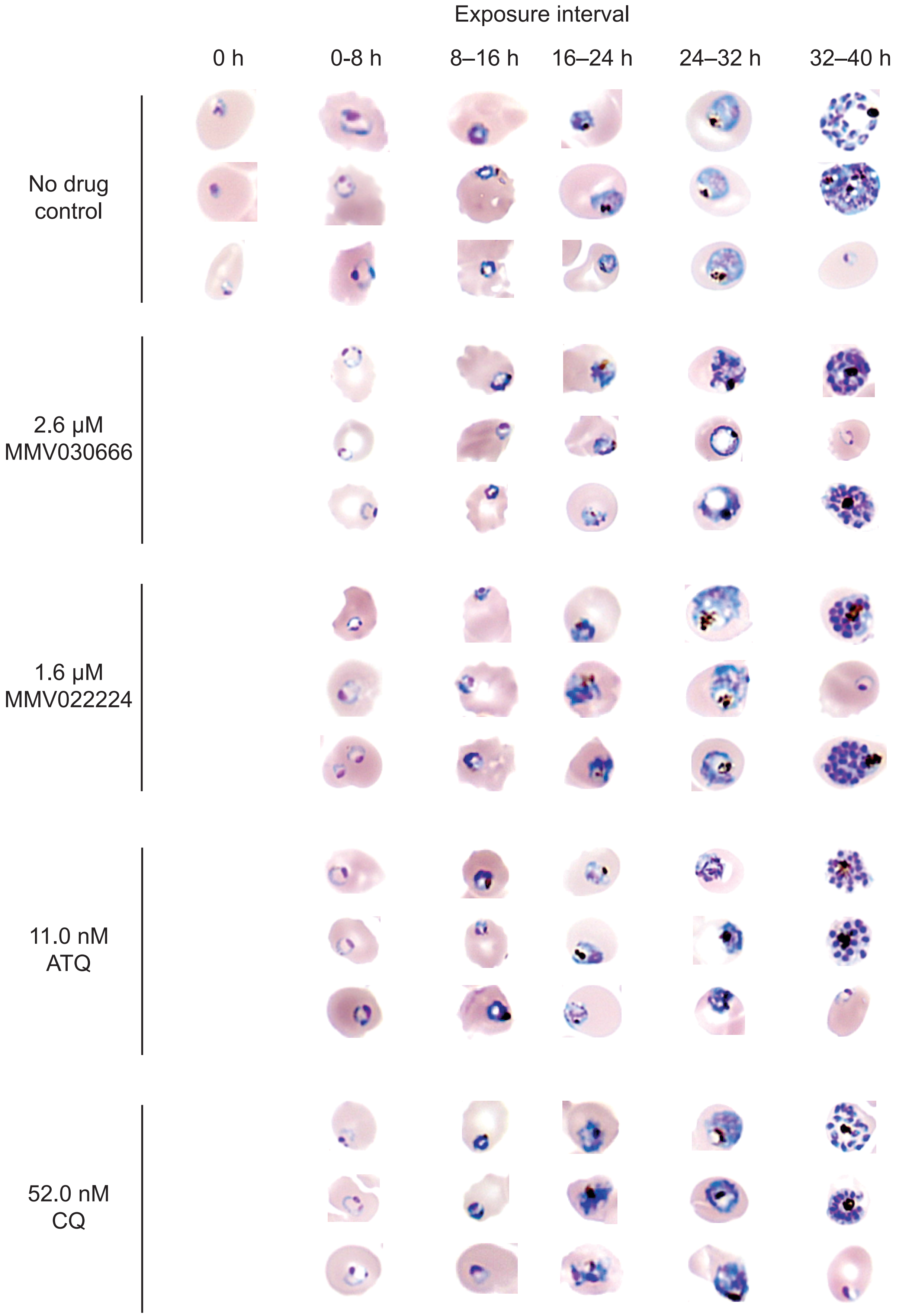

Figure S4 (related to Figure 2 and Figure 4). Microscopical studies confirm the stage specificity profiles of MMV030666 and MMV022224, using ATQ and CQ as controls. Synchronized parasites were exposed to 3× their lowest IC_{50}^{8h} at the indicated life stages, and were assessed at the end of each exposure. ATQ: atovaquone, CQ: chloroquine.
