# Supplementary material for: Combining Stage Specificity and Metabolomic Profiling to Advance Antimalarial Drug Discovery
Source: Cell Chem Biol. 2020 Feb 20;27(2):158–171.e3. doi: 10.1016/j.chembiol.2019.11.009 (PMC7031696; doi:10.1016/j.chembiol.2019.11.009)
Supplement: Document S2. Article plus Supplemental Information [file mmc3.pdf]

Article

Cell Chemical Biology

Combining Stage Specificity and Metabolomic Profiling to Advance Antimalarial Drug Discovery

Highlights

- Timing of peak activity during life cycle informs on mode of action of antimalarials
- Results differentiated modes of action of piperaquine, lumefantrine, and atovaquone
- Experimental compounds targeted previously unexplored druggable pathways
- Characterized ring-active compounds and new heme or mitochondrial inhibitors

Authors

James M. Murithi, Edward S. Owen, Eva S. Istvan, ..., Manuel Llinás, David A. Fidock, Manu Vanaerschot

Correspondence

df2260@cumc.columbia.edu (D.A.F.), manu.vanaerschot@gmail.com (M.V.)

In Brief

Murithi et al. designed a high-throughput assay to profile the *Plasmodium falciparum* asexual blood stage parasites against which antimalarial candidates are maximally active. These data combined with metabolomics fingerprinting provide valuable insights into the mode of action of clinical and experimental antimalarials, and helped prioritize compounds for further development.

Murithi et al., 2020, Cell Chemical Biology *27*, 158–171
February 20, 2020 © 2019 The Author(s). Published by Elsevier Ltd.
https://doi.org/10.1016/j.chembiol.2019.11.009

Cell Chemical Biology
Article

Combining Stage Specificity and Metabolomic Profiling to Advance Antimalarial Drug Discovery

James M. Murithi,[1] Edward S. Owen,[2] Eva S. Istvan,[3] Marcus C.S. Lee,[4] Sabine Ottilie,[5] Kelly Chibale,[6,7] Daniel E. Goldberg,[3] Elizabeth A. Winzeler,[5] Manuel Llinás,[2,8,9] David A. Fidock,[1,10,*] and Manu Vanaerschot[1,11,*]
[1]Department of Microbiology and Immunology, Columbia University Irving Medical Center, New York, NY 10032, USA
[2]Department of Biochemistry and Molecular Biology, Pennsylvania State University, University Park, PA 16802, USA
[3]Department of Medicine, Division of Infectious Diseases, and Department of Molecular Microbiology, Washington University School of Medicine, Saint Louis MO 63130, USA
[4]Parasites and Microbes Programme, Wellcome Sanger Institute, Wellcome Genome Campus, Hinxton, Cambridgeshire CB10 1SA, UK
[5]School of Medicine, University of California San Diego (UCSD), La Jolla, CA 92093, USA
[6]Drug Discovery and Development Centre (H3D), University of Cape Town, Rondebosch 7701, South Africa
[7]South African Medical Research Council Drug Discovery and Development Research Unit, Department of Chemistry & Institute of Infectious Disease and Molecular Medicine, University of Cape Town, Rondebosch 7701, South Africa
[8]Department of Chemistry, Pennsylvania State University, University Park, PA 16802, USA
[9]Huck Center for Malaria Research, Pennsylvania State University, University Park, PA 16802, USA
[10]Division of Infectious Diseases, Department of Medicine, Columbia University Irving Medical Center, New York, NY 10032, USA
[11]Lead Contact
*Correspondence: df2260@cumc.columbia.edu (D.A.F.), manu.vanaerschot@gmail.com (M.V.)
https://doi.org/10.1016/j.chembiol.2019.11.009

SUMMARY

We report detailed susceptibility profiling of asexual blood stages of the malaria parasite *Plasmodium falciparum* to clinical and experimental antimalarials, combined with metabolomic fingerprinting. Results revealed a variety of stage-specific and metabolic profiles that differentiated the modes of action of clinical antimalarials including chloroquine, piperaquine, lumefantrine, and mefloquine, and identified late trophozoite-specific peak activity and stage-specific biphasic dose-responses for the mitochondrial inhibitors DSM265 and atovaquone. We also identified experimental antimalarials hitting previously unexplored druggable pathways as reflected by their unique stage specificity and/or metabolic profiles. These included several ring-active compounds, ones affecting hemoglobin catabolism through distinct pathways, and mitochondrial inhibitors with lower propensities for resistance than either DSM265 or atovaquone. This approach, also applicable to other microbes that undergo multiple differentiation steps, provides an effective tool to prioritize compounds for further development within the context of combination therapies.

INTRODUCTION

Malaria caused by the protozoan parasite *Plasmodium falciparum* (Pf) remains a major public health menace, especially in young children in sub-Saharan Africa (WHO, 2018). When an individual is bitten by a *Plasmodium*-infected mosquito, the parasite first replicates in hepatocytes and then initiates ~48-h cycles of red blood cell (RBC) infection. In these RBCs, the parasite develops inside a parasitophorous vacuole, progressing from a ring into a highly metabolically active trophozoite and then a multinucleated schizont that yields 8–24 merozoites generated through asexual replication. Upon egress from the lysed host RBC, these merozoites infect new RBCs, with parasites capable of infecting up to 10%–20% of RBCs in an immunologically naive host (Phillips et al., 2017).

Chemotherapy remains a major pillar in the fight against malaria, alongside vector control, diagnosis, and access to treatment. The former first-line antimalarials chloroquine and sulfadoxine-pyrimethamine mainly affect trophozoites by inhibiting the hemoglobin catabolism pathway that provides nutrients for the parasite and the folate biosynthesis pathway that delivers the building blocks for DNA synthesis, respectively (Blasco et al., 2017). KAI407, a phosphatidylinositol 4-kinase (PI4K) inhibitor, is one of the more recent candidate antimalarials that specifically inhibit schizont development (McNamara et al., 2013). These drugs mostly target trophozoites and schizonts, which sequester in the microvasculature (Miller et al., 2002). Compounds targeting ring stages, which circulate throughout the blood stream, are desirable to prevent further vasculature blockage. Artemisinins were the first clinical antimalarials with ring-stage activity, and artemisinin-based combination therapies have proven effective in reducing malaria death and case load (WHO, 2018). However, parasites resistant to artemisinins and their partner drugs have emerged and are now undermining malaria control (Menard and Dondorp, 2017; Ross and Fidock, 2019). The discovery of antimalarials that hit novel targets and are active against multiple asexual blood stages, including rings, is thus of paramount importance.

Thousands of antimalarials with submicromolar potency have been identified in high-throughput whole-cell screens (Antonova-Koch et al., 2018; Delves et al., 2018; Gamo et al., 2010; Guiguemde et al., 2010; Plouffe et al., 2008; Raphemot et al., 2015; Wu et al., 2015), but target identification forms a major

 © 2019 The Author(s). Published by Elsevier Ltd.
This is an open access article under the CC BY license (http://creativecommons.org/licenses/by/4.0/).

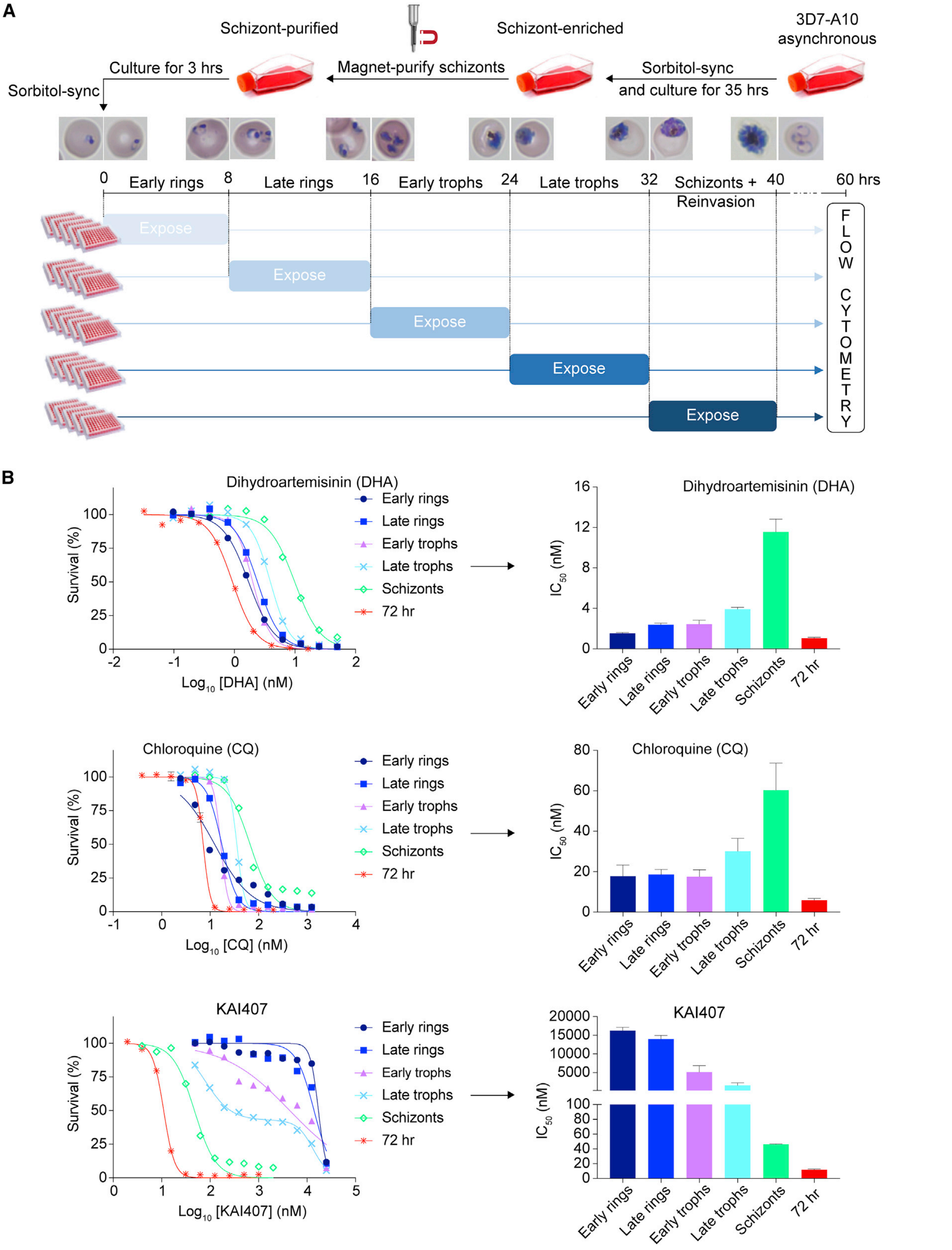

(legend on next page)

bottleneck for their further development into leads with increased target binding, selectivity, and whole-cell activity (Okombo and Chibale, 2017). Metabolomic analysis of biochemical pathways affected upon compound exposure recently identified the mode of action of various candidate antimalarials from the Medicines for Malaria Venture Malaria Box (Allman et al., 2016), and is a valuable tool to interrogate new screening hits. Combining this approach with other phenotypic assays can help explore the activity profile and therapeutic potential of candidate antimalarials.

The Malaria Drug Accelerator (MalDA) consortium aims to identify new antimalarial leads through *in vitro* phenotypic screens and the identification of novel assayable targets (Antonova-Koch et al., 2018; Cowell et al., 2018). Within this context, we developed an assay that compares the stage-specific susceptibility of Pf asexual blood stage parasites and combined this with metabolomic profiling.

RESULTS

We designed a medium-throughput *in vitro* assay to quantitatively assess the susceptibility of the distinct stages of Pf intra-erythrocytic development. Highly synchronized 3D7-A10 parasites (that have an accelerated 40-h asexual blood stage cycle) were exposed to a range of compound concentrations for 8 h during the early ring, late ring, early trophozoite, late trophozoite, and schizont stages (Figure 1A). Assays were performed in 96-well plates, with a maximum in-well DMSO concentration of 0.35%. Cultures were continued to allow parasites to further develop in the absence of compound, extending through to invasion of new RBCs and development until the trophozoite stage. The total assay duration was 60 h. Parasites were stained with SYBR green and Mitotracker Deep Red and quantified by flow cytometry. Half-maximal inhibitory concentrations (IC_{50}) were derived by non-linear regression analyses of the dose-response data. The IC_{50} value based on these 8-h exposures at specific asexual blood stages is referred to as the $IC_{50}{}^{8h}$, while the IC_{50} calculated from the standard 72-h exposure assay is the $IC_{50}{}^{72h}$.

Light microscopy confirmed that the different periods of exposure corresponded to the different developmental stages and showed that the 32- to 40-h time point spanned schizont development, parasite egress, and reinvasion (Figure 1A), indicating that all asexual blood stages were profiled. The assay was further validated by the stage-specific susceptibility profiles of dihydroartemisinin, chloroquine, and KAI407, which showed the expected peak activity on early rings, rings and trophozoites, and schizonts, respectively (Blasco et al., 2017; Zhang et al., 1986) (Figure 1B). The 35-fold difference in $IC_{50}{}^{8h}$ between schizonts and late trophozoites for KAI407 (Table S1) highlighted the tight synchronization of parasites that is crucial for this assay.

The asexual blood stage susceptibility profile was determined for a set of 36 compounds that included licensed drugs, candidate antimalarials, compounds with a known target, and various screening hits (profiles of compounds are shown in Figures 2, 3, 4, and 5, simplified molecular input line entry system descriptions for compounds are listed in Table S2, and structures of compounds are displayed in Figures S1 and S2). Hits were selected from screens previously performed by the MalDA consortium (see Table S2 references) and prioritized based on their potency, chemical diversity, and unknown mode of action. Licensed antimalarial drugs and additional previously published preclinical compounds were included to provide more insights into their mode of action or to serve as a reference.

First, compounds were classified based on their timing of peak activity, defined as the asexual blood stage at which the compounds showed the lowest $IC_{50}{}^{8h}$ values. This identified compounds with peak activity during (1) all rings and trophozoites, (2) all rings, (3) all trophozoites, (4) all trophozoites and schizonts, (5) late trophozoites, and (6) schizonts (Figure 6). When compounds were classified by their overall activity profile based on identifying the specific stages that showed $IC_{50}{}^{8h}$ values <1 μM (Figure S3; Table S1), seven active classes were identified: compounds active on (1) all asexual blood stages, (2) all rings and trophozoites, (3) late rings and all trophozoites, (4) all trophozoites and schizonts, (5) late trophozoites and schizonts, (6) only late trophozoites, and (7) only schizonts. Fosmidomycin, a moderately potent inhibitor of Pf isoprenoid biosynthesis (Jomaa et al., 1999), as well as the hit compounds MMV000787, MMV019017, MMV020746, MMV022478, and MMV665939, showed $IC_{50}{}^{8h}$ values >1 μM at all tested stages and therefore did not match any of these groups (Table S1).

The clinical antimalarials dihydroartemisinin, chloroquine, piperaquine, and lumefantrine showed little variation in $IC_{50}{}^{8h}$ values throughout the ring and trophozoite stages, and were consequently classified in the group with peak activity at ring and trophozoite stages. Although chloroquine, piperaquine, and lumefantrine $IC_{50}{}^{8h}$ values were similar for ring and trophozoite stages, survival curves for early rings were less steep than those for late rings and trophozoites (Figures 1 and 2). DSM265 and atovaquone, which are inhibitors of pyrimidine synthesis and the mitochondrial electron transport chain, respectively (Figure 5A), showed peak activity specifically during late trophozoite stages (Figures 5B and 6). These mitochondrial inhibitors also displayed a biphasic survival curve at the early trophozoite and schizont stages that was not observed at other stages (Figure 5B; Table S1).

MMV000442, MMV006455, MMV007181, and MMV665971 showed incomplete killing at all asexual blood stages, with evidence of initial growth inhibition at lower concentrations followed by demonstrably better growth at higher concentrations in the early and late ring stages (Figures 3 and 4). This incomplete killing was not observed in the 72-h exposure survival curves for these compounds (Figures 3 and 4). Aqueous solubility experiments for MMV000442, MMV006455, and MMV007181 indicated a solubility >100 μM (Table S3), well above the highest concentration used in the stage specificity assay.

Figure 1. Experimental Design for Asexual Blood Stage Specificity Profiling of Antimalarials and Profiles of Reference Drugs

(A) Synchronized parasites were exposed for 8 h at the stages indicated. Survival at 60 h post-invasion was assessed by flow cytometry.

(B) Unique stage specificity profiles of chloroquine, dihydroartemisinin, and KAI407. Bar plots indicate the $IC_{50}{}^{8h}$ when parasites were exposed only during the early ring, late ring, early trophozoite, late trophozoite, or schizont stage, with error bars showing the standard error of the mean based on at least three independent repeats. KAI407, a PI4K inhibitor. All data are available in Table S1.

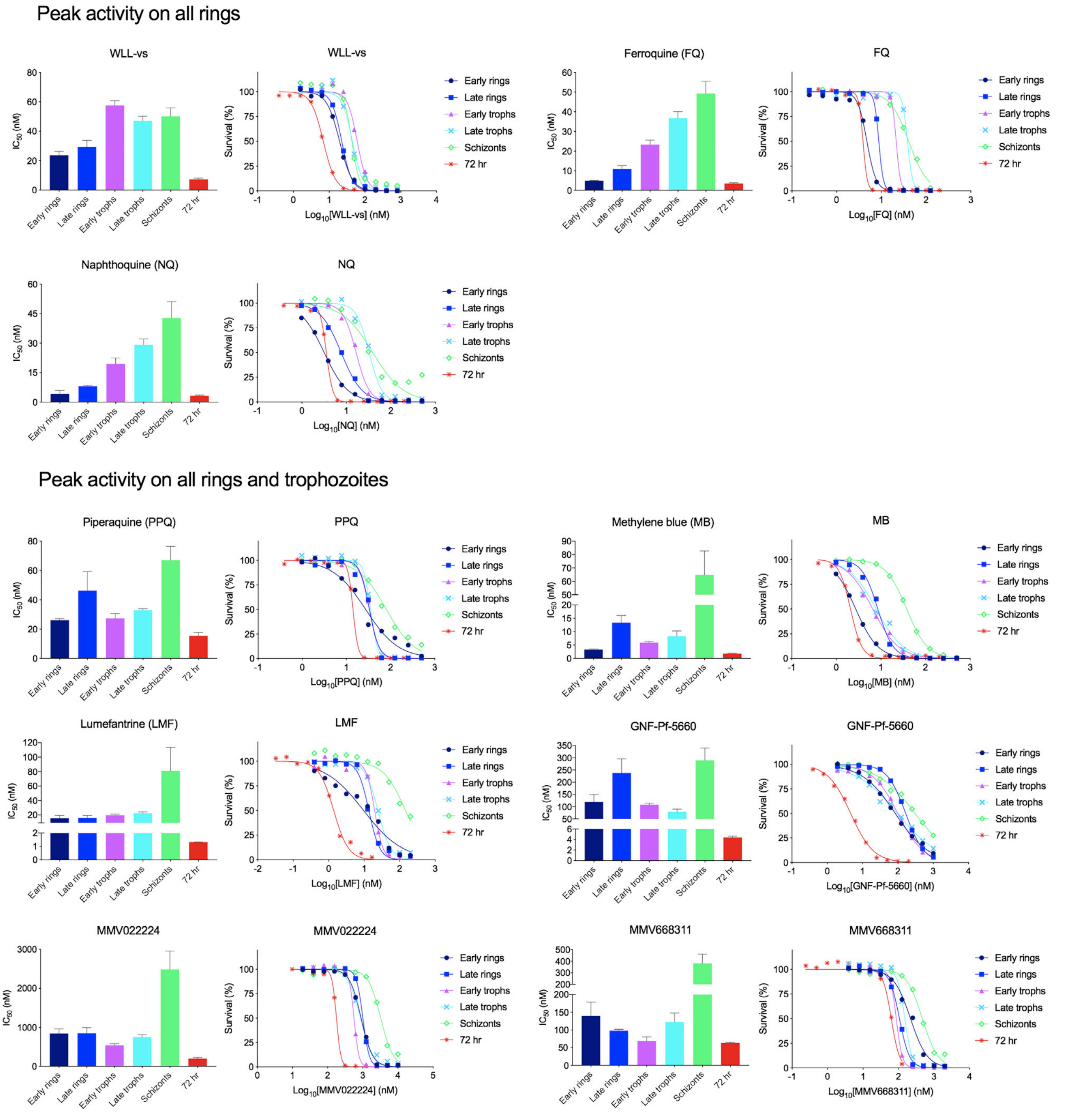

Figure 2. Detailed Asexual Blood Stage Susceptibility Profiles for Antimalarials with Peak Activity on All Rings or All Rings and Trophozoites
Data for chloroquine and dihydroartemisinin can be found in Figure 1. Bar graphs indicate mean IC_{50}^{8h} values, whereas survival graphs show the most representative curves from independent repeats. Error bars indicate the standard error of the mean based on >3 independent repeats. Data are summarized in Table S1.

To further examine whether the compound stage specificity profiles that we identified correlated with their mode of action, we examined the metabolic profile of 33 compounds (Figure 7; Table S4). These consisted of 27 newly assayed compounds, plus another 6 (chloroquine, DSM265, MMV000248, MMV006455, MMV019017, and KAE609) for which data were already available (Allman et al., 2016). In these experiments, we exposed trophozoite-infected RBCs to 10 × IC_{50}^{72h} concentrations and then subjected parasite extracts to mass spectrometry-based metabolomic profiling (Allman et al., 2016).

Across all 33 compounds, we obtained quantitative data for 195 metabolites that represent major metabolic pathways, including but not limited to pyrimidine and purine synthesis, hemoglobin catabolism, folate biosynthesis, central carbon

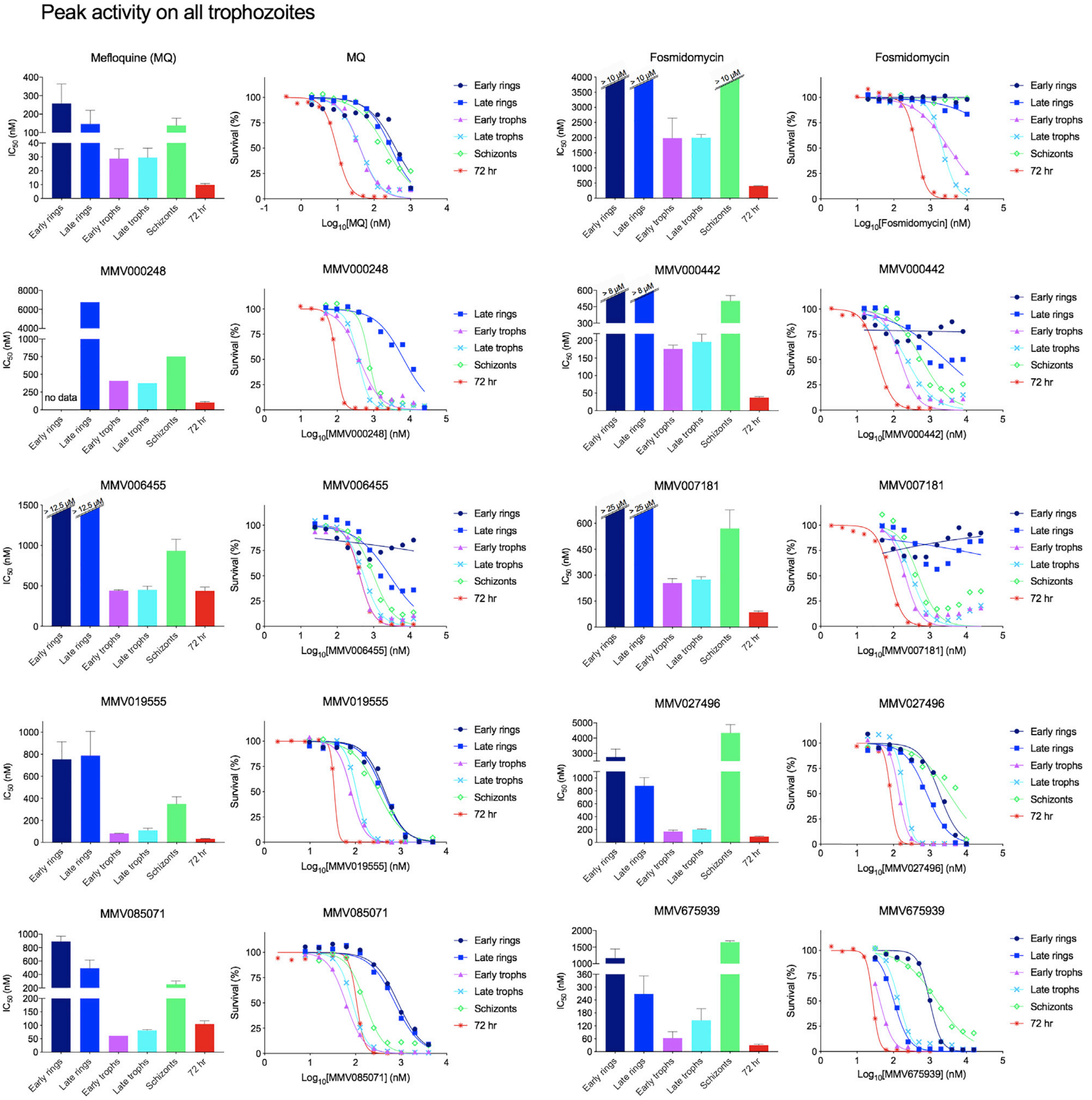

Figure 3. Detailed Asexual Blood Stage Susceptibility Profiles for Antimalarials with Peak Activity on All Trophozoites

Bar graphs indicate mean IC_{50}^{8h} values, whereas survival graphs show the most representative curves from independent repeats. Error bars indicate the standard error of the mean based on >3 independent repeats. Data are summarized in Table S1.

metabolism, glycolysis, and redox metabolism (Table S4). Based on these metabolic profiles, compounds were hierarchically clustered via Ward clustering based on Pearson correlation coefficients to identify related metabolic signatures (Figure 7).

Several established metabolic signatures were observed among the analyzed compounds. Mitochondrial electron transport chain disruption is linked to inhibition of dihydroorotate dehydrogenase (DHODH) and cytochrome bc_1 (CytBC1), leading to increases in the pyrimidine precursors dihydroorotate and N-carbamoyl-L-aspartate (Allman et al., 2016). This metabolic signature was observed for DSM265 and ATQ, which respectively inhibit DHODH and CytBC1, as well as MMV000787, MMV021735, and MMV030666, for which the mode of action was previously unknown (Figure 7; Table S4).

We performed resistance selections with MMV021735, MMV030666, and MMV000787 to compare their propensity for resistance with that of DSM265 and atovaquone, which have a relatively low minimum inoculum for resistance of 2×10^6 and

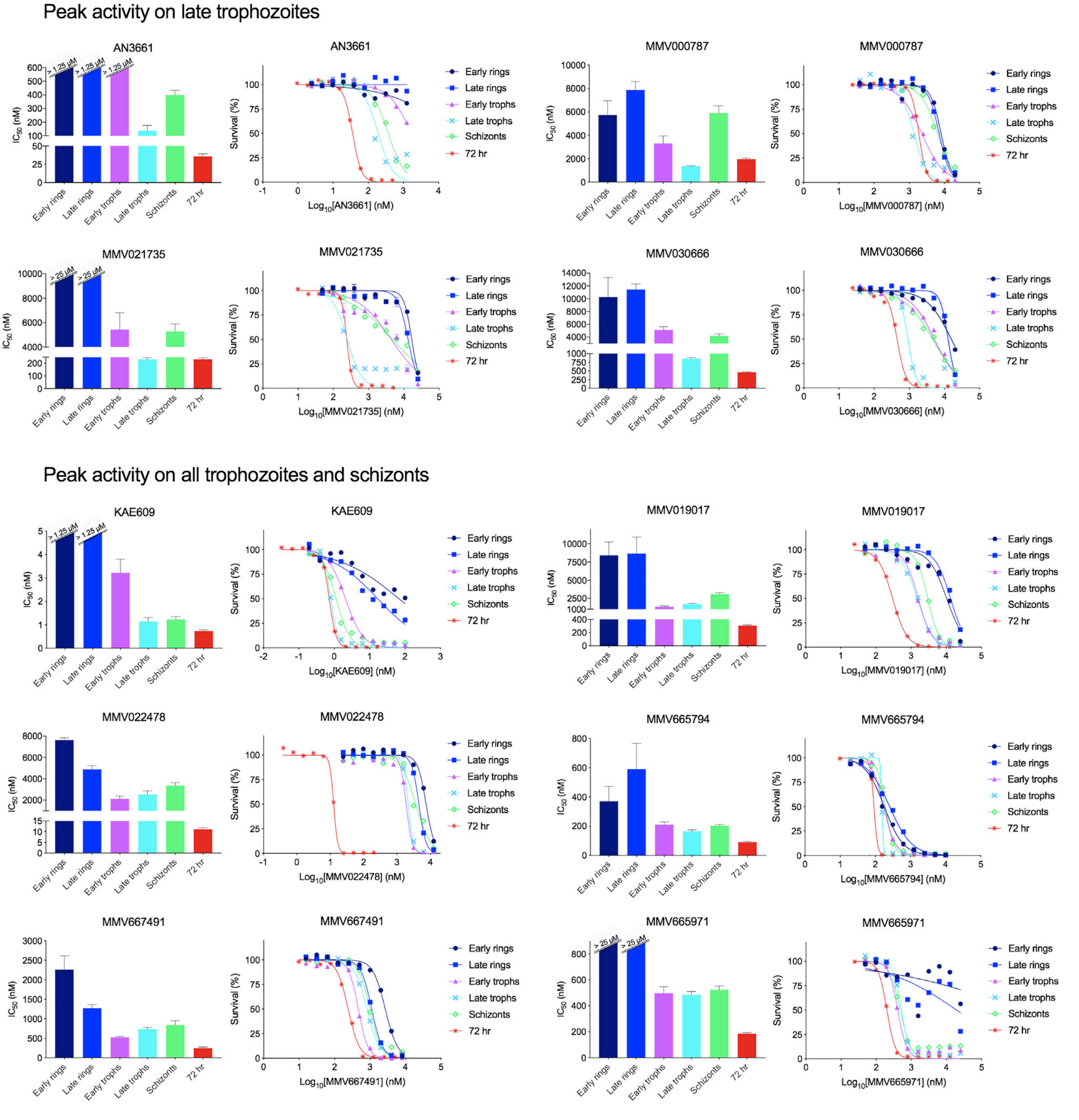

Figure 4. Detailed Asexual Blood Stage Susceptibility Profiles for Antimalarials with Peak Activity on Late Trophozoites, or on All Trophozoites and Schizonts

Data for DSM265 and atovaquone, both compounds with peak activity at the late trophozoite stage, can be found in Figure 5. Bar graphs indicate mean IC_{50}^{8h} values, whereas survival graphs show the most representative curves from independent repeats. Error bars indicate the standard error of the mean based on >3 independent repeats. Data are summarized in Table S1.

2×10^7parasites, respectively, when using $3 \times IC_{50}^{72h}$ drug concentrations (Phillips et al., 2015). Selections involving continuous exposure of 1×10^9 Dd2-B2 parasites to a $3.5 \times IC_{50}^{72h}$ concentration of MMV000787, or intermittent drug pulsing in which parasites were exposed for several days at a time to $6 \times IC_{50}^{72h}$ concentrations of MMV000787 for 5 months, did not result in MMV000787-resistant parasites. For MMV021735, exposing 5×10^8 3D7-A10 parasites to $3 \times IC_{50}^{72h}$ concentrations in triplicate failed to yield resistant parasites. Exposing 5×10^8 3D7-A10 or Dd2-B2 parasites to $3 \times IC_{50}^{72h}$ concentrations of MMV030666 also failed to yield resistance. A ramping selection with 3D7-A10 parasites starting at $1 \times IC_{50}^{72h}$ and

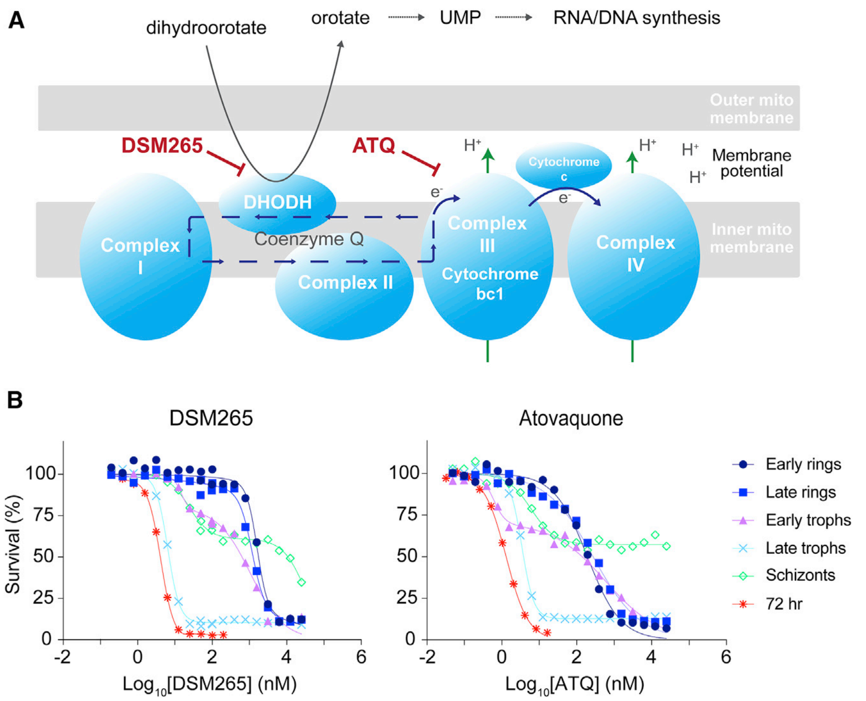

Figure 5. Late Trophozoites Are the Most Susceptible Stage to DSM265 and Atovaquone that Inhibit Pyrimidine Biosynthesis and the Mitochondrial Electron Transport Chain, Respectively

(A) Overview of the pyrimidine biosynthesis and the mitochondrial electron transport chain pathways. DSM265 inhibits DHODH, whereas atovaquone inhibits cytochrome bc1 (Goodman et al., 2017).

(B) Stage specificity profiles for DSM265 and atovaquone. IC_{50}^{8h} values for (B) are available in Table S1.

gradually increasing to 1.8 × IC_{50}^{72h} over the course of 3 months also did not produce resistance. These data indicate that MMV000787, MMV21735, and MMV030666 have minimum inocula of resistance well above 5 × 10^8 parasites.

Peptide decreases commonly linked with inhibition of hemoglobin endocytosis and/or catabolism within the digestive vacuole were also observed across multiple antimalarial compounds (Figure 7; Table S4). This metabolic signature of decreased peptide levels (HVDD, PVNF, PEEK, PEE, DLS, SDL, SID, DLH, LD, PE, PD, SD, VD, and EV) was particularly pronounced for the compounds MMV022478, MMV019555, MMV667491, MMV030666, MMV000248, MMV006455, MMV007181, MMV020746, MMV 000442, GNF-Pf-5660, KAE609, and MMV019017. Of these, MMV006455, MMV019017, and KAE609 also possessed increased levels of the deoxyribonucleotides dAMP and dTMP and decreased levels of cAMP. KAE609, and MMV019017 also showed decreased nucleoside di- and tri-phosphate levels (GDP, UDP, GTP, dATP, dGTP/ATP, and dUTP), which have previously been identified as a signature of inhibiting the Na^+/H^+-dependent ATPase PfATP4 (Allman et al., 2016). The hemozoin inhibitor chloroquine did not show the expected strong hemoglobin catabolism signature, but instead showed a more modest decrease in peptide levels and clustered with the PfATP4-inhibitor KAE609.

Interestingly, the metabolic profile for MMV030666 indicated perturbation of both the mitochondrial electron transport chain and hemoglobin catabolism. MMV022224 induced increased levels of peptides, a profile that has not been observed before. Because of the peculiar profiles of these compounds, they were selected for an additional study in which synchronized parasites were exposed to 3× the IC_{50}^{8h} of the most sensitive life stage at 8-h intervals, similar to the stage specificity assay, and cell morphology was assessed by microscopy at the end of each interval (Figure S4). This showed MMV030666-exposed parasites to be most susceptible during the late trophozoite stage, as demonstrated by their bloated digestive vacuoles. This phenotype is characteristic of hemoglobin catabolism perturbation (Ross et al., 2018), and is consistent with the metabolomics data. MMV022224-exposed parasites proved to be affected mostly during early and late trophozoite stages, without displaying swollen vacuoles. The health of ring-stage parasites, which showed similar IC_{50}^{8h} values as trophozoites for MMV022224, was harder to microscopically evaluate due to their smaller size.

Mefloquine, naphthoquine, piperaquine, methylene blue, MMV675939, dihydroartemisinin, WLL-vs, MMV668311, ferroquine, and MMV085071 did not induce major changes within the set of metabolites detected in our study and, therefore, clustered in the low fold change group (Figure 7; Table S4).

DISCUSSION

Herein we report the results of Pf asexual blood stage susceptibility assays that compared the susceptibility of early rings, late rings, early trophozoites, late trophozoites, and schizonts, for a set of 36 clinical and experimental antimalarials. These studies, which exposed each tightly synchronized stage for 8 h and assessed overall parasite growth at the same 60-h time point (Figure 1A), extend earlier experimental designs that assessed activity on a subset of stages or did not include wash-offs to restrict exposure to each stage (Duffy and Avery, 2017; Wilson et al., 2013). Because compounds are washed out after each exposure moment and parasites are allowed to continue to grow in absence of compound until the end of the assay, the stage specificity assay quantifies the cytotoxic (killing) effect of compounds. The IC_{50}^{8h} values are therefore in essence stage-specific half maximal lethal doses (Paguio et al., 2011). This contrasts with the IC_{50}^{72h} values that are determined in assays that expose parasites continuously to compounds and measure the cytostatic (growth inhibitory) effect of compounds. Our results were combined with metabolomic profiling of the cellular pathway perturbations caused by these compounds, as an exploratory approach to identify common or unique profiles among the tested antimalarials. Classification of compounds according to the timing of their peak activity revealed a remarkable variety of profiles among both clinical and experimental compounds

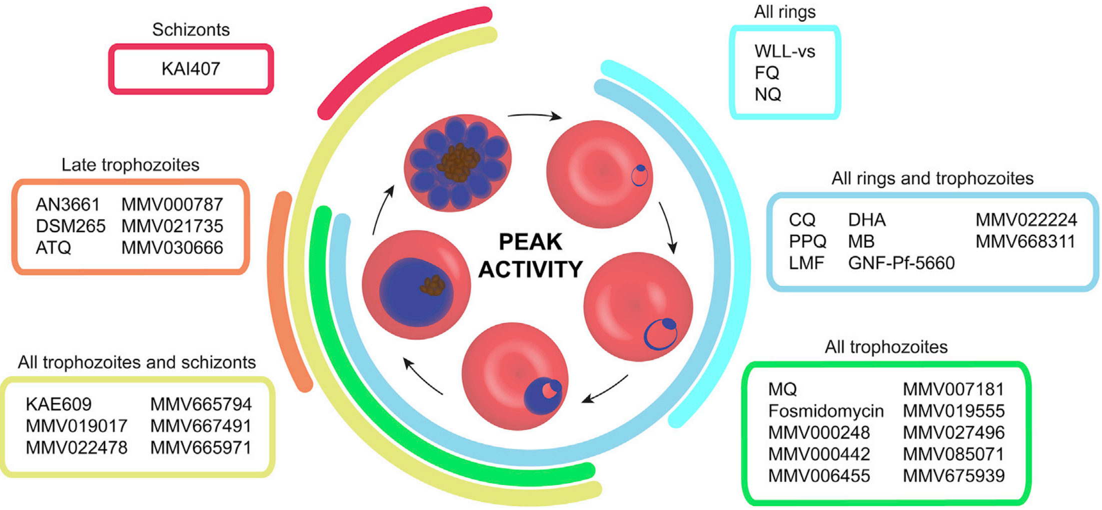

Figure 6. Stage of Peak Activity for Clinical and Experimental Antimalarials

Peak activity illustrates the period when the parasite was most susceptible to the tested compounds. MMV020746 and MMV665939 were omitted as their IC_{50}^{8h} values were >10 μM. All data are available in Table S1 and Figures 1, 2, 3, 4, and 5. ATQ, atovaquone; CQ, chloroquine; DHA, dihydroartemisinin; FQ, ferroquine; LMF, lumefantrine; MB, methylene blue; MQ, mefloquine; NQ, naphthoquine; PPQ, piperaquine.

(Figure 6). As examples, the inhibitors DSM265 and atovaquone (which target DHODH and CytBC1, respectively) showed activity against late trophozoites only, and the PI4K inhibitor KAI407 showed activity against only schizonts, in good agreement with earlier studies (McNamara et al., 2013; Painter et al., 2010; Phillips et al., 2015) (Figures 1 and 5; Table S1).

Compounds with different chemical scaffolds that are known to target the same or related pathways showed similar stage specificity and metabolic profiles. This was especially apparent for atovaquone and DSM265 that act on related mitochondrial processes (Figure 5A). These agents also shared similar killing dynamics, with a monophasic survival curve for the highly sensitive late trophozoites and biphasic curves for early trophozoites and schizonts (Figure 5B). Of note, when parasite survival was assessed using only the SYBR green signal, and not the Mitotracker signal, we observed the same killing dynamics for atovaquone and DSM265. This likely reflects a dual purpose of the mitochondria of maintaining their membrane potential through the mitochondrial electron transport chain, required for the production of ATP, and enabling pyrimidine biosynthesis through DHODH (Figure 5A). Inhibition of DHODH by DSM265 will not only affect pyrimidine biosynthesis but also the recycling of ubiquinone, which is crucial for the parasite to maintain its mitochondrial membrane potential. Likewise, inhibition of CytB by atovaquone will not only directly affect the mitochondrial membrane potential, but also the recycling of ubiquinone and therefore the function of DHODH. DHODH and CytB are thus two distinct drug targets that are functionally linked. In accordance, DSM265 and atovaquone show the same stage specificity profile. Pyrimidines are most needed in late trophozoites when DNA synthesis peaks, allowing the production of daughter merozoites during schizogony (Cassera et al., 2011). Without pyrimidines, late trophozoites would not be able to develop into functional schizonts, resulting in a low IC_{50}^{8h} and a smooth monophasic killing curve in late trophozoites (Figure 5B). In early trophozoites and schizonts, the dependency on pyrimidines is lower but a functional mitochondrial membrane potential would still appear to be vital for the many ongoing biological processes, leading to a biphasic response in which the first shift relates to pyrimidine biosynthesis and a second shift relates to the mitochondrial membrane potential. Early and late rings showed a monophasic response with high IC_{50}^{8h} values, reflecting a parasite growth phase when pyrimidine biosynthesis and mitochondrial activity appear to be minimal. Atovaquone inhibition through membrane potential disruption was relatively ineffective in our 8-h exposure model, illustrating the need for longer compound exposure for mitochondrial electron transport chain inhibitors (Gomez-Lorenzo et al., 2018; Painter et al., 2010). Importantly, incomplete killing by atovaquone and DSM265 was observed in all stages, matching previous data from recrudescence-based assays that showed atovaquone to be a slow and incomplete killer (Linares et al., 2015; Sanz et al., 2012).

Of note, the late trophozoite stage specificity profiles for ATQ and DSM265 are consistent with the timing of expression of their targets: *cytb* expression peaks during the late trophozoite stage, whereas maximal expression of *dhodh* spans early to late trophozoite stages (Painter et al., 2018). The same holds true for KAE609, which targets PfATP4: transcription of *pfatp4* peaks at the early trophozoite stage (Painter et al., 2018), consistent with KAE609 being inactive against rings yet active against early trophozoites and later stages. Interestingly, *pi4k*, which encodes the target of KAI407, is transcribed at fairly stable levels without showing a clear peak at any stage (Painter et al., 2018). The schizont-specific activity profile of KAI407 may be determined by the availability of substrates that interact at this stage with PI4K.

These assays also differentiated the mode of action of chloroquine, piperaquine, and mefloquine, which share a core 4-aminoquinoline ring structure. Piperaquine essentially consists of two molecules of chloroquine connected by a central linker. Chloroquine and piperaquine are generally thought to act at the highly metabolically active trophozoite stage by inhibiting

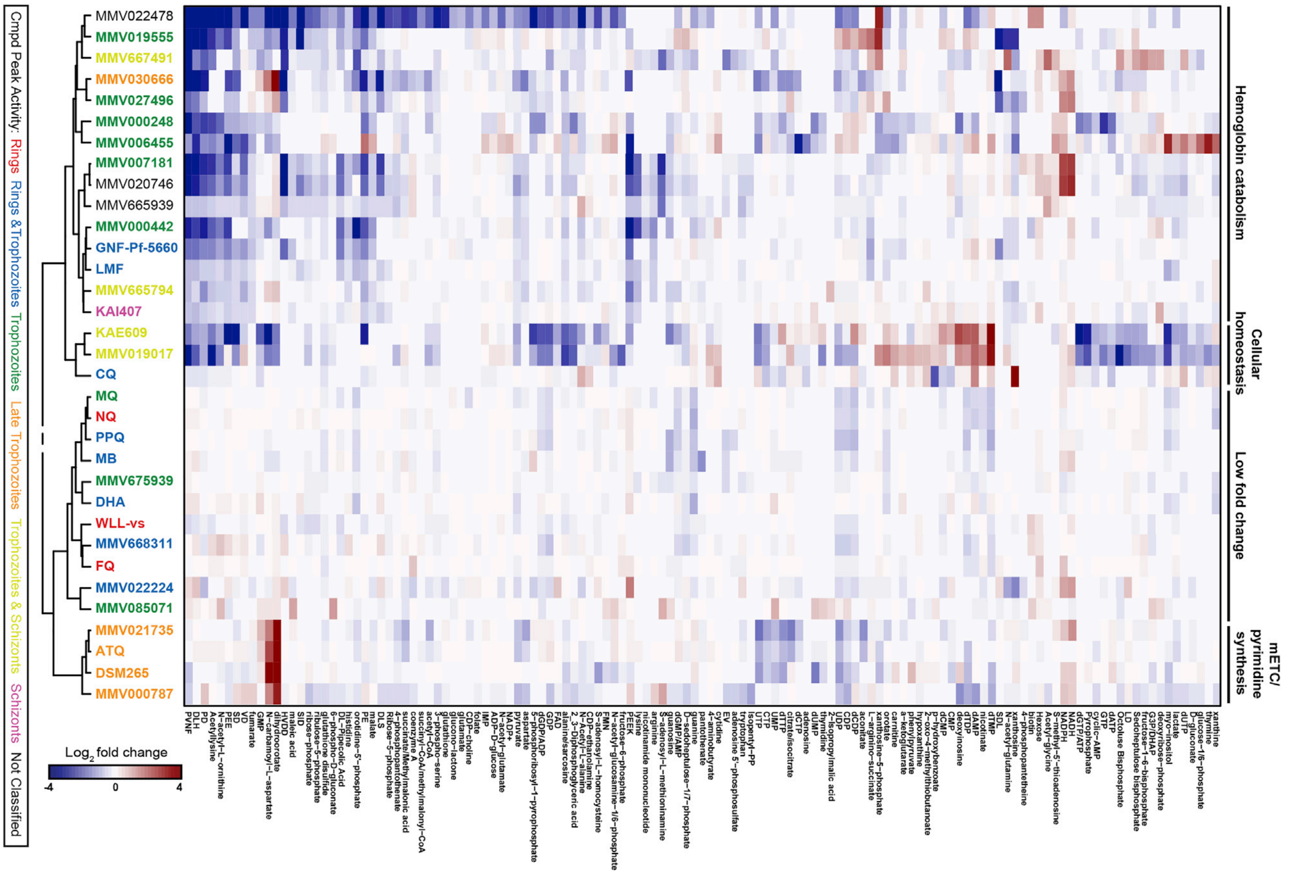

Figure 7. Metabolic Profiling of Compounds Identified Cellular Processes Targeted by Compounds

Compounds were clustered based on hydrophilic metabolite response to all measured metabolites (all data available in Table S4). Compounds are listed only if they showed a >2-fold change ($log_2 > 1$) in metabolite levels compared with untreated controls in at least one of the treated samples. Compounds are color-coded based on peak activity as shown in Figure 6. Metabolite data for chloroquine, DSM265, MMV000248, MMV006455, MMV019017, and KAE609 were sourced from (Allman et al., 2016). Data for all other 27 compounds were generated in this study. ATQ, atovaquone; Cmpd, compound; CQ, chloroquine; DHA, dihydroartemisinin; FQ, ferroquine; LMF, lumefantrine; MB, methylene blue; mETC, mitochondrial electron transport chain; MQ, mefloquine; NQ, naphthoquine; PPQ, piperaquine.

the biomineralization of free heme, released during hemoglobin digestion, into hemozoin, thereby causing a buildup of toxic free heme or heme-drug adducts (Blasco et al., 2017). Both chloroquine and piperaquine showed a similar stage specificity profile when the error margin is taken into account, and exerted potent growth inhibition in early ring stages. This would suggest that hemoglobin catabolism begins even in early rings, before the formation of the digestive vacuole inside which the bulk of hemozoin is generated. This inference is supported by a previous report (Zhang et al., 1986) and studies that detected hemoglobin uptake (Elliott et al., 2008) and activity of falcipains (required for hemoglobin digestion [Xie et al., 2016]) in very early rings. Notably, early rings showed a flatter slope of the dose-dependent curve than late rings and trophozoites, indicating different growth inhibitory dynamics (Figures 1 and 2). Metabolic perturbation profiles, nonetheless, revealed a strikingly different profile for chloroquine and piperaquine (Figure 7; Table S4). Chloroquine induced various perturbations that were not observed under piperaquine pressure, such as >2-fold increased levels of dAMP, dUTP, cytidine, xanthosine, and N-acetyl-lysine, decreased *p*-hydroxybenzoate levels, and decreased peptide levels that are characteristic for hemoglobin catabolism inhibition. Some of these metabolic changes in chloroquine-exposed parasites, such as the increased dAMP levels, caused chloroquine to metabolically cluster with the PfATP4 inhibitor KAE609 and other compounds that cause an overall disturbance in cellular homeostasis. This clustering, however, is based on rather modest changes and should be interpreted with caution. Piperaquine metabolically clustered with other compounds that induced an overall low differential fold change (Figure 7; Table S4). The only notable changes were ≥2-fold decreased levels of dCDP, dTMP, guanosine, and guanine. This suggests that piperaquine might have an additional mode of action beyond inhibition of hemozoin formation that perturbs purine and pyrimidine metabolism.

Mefloquine, an arylamino alcohol that also shares a quinoline ring, was earlier reported to inhibit hemozoin formation in parasites at a lower level than chloroquine (Combrinck et al., 2013), possibly because of reduced mefloquine accumulation in the digestive vacuole. Earlier studies examining mefloquine and its

relationship to the primary resistance determinant PfMDR1 (located on the membrane of the digestive vacuole) suggested that mefloquine acts primarily outside the digestive vacuole (Veiga et al., 2016). The difference in mode of action between mefloquine and chloroquine is also reflected in their stage specificity and metabolomics profiles, with mefloquine showing peak activity only in trophozoites and clustering separately from other compounds affecting hemoglobin catabolism (Figure 7). These data further support the notion that the target of mefloquine is presumably located outside of the digestive vacuole, affecting the parasite in ways that could not be detected by our metabolomics study.

The clinical antimalarial lumefantrine displayed peak activity during both rings and trophozoites, similar to chloroquine and piperaquine but different from the trophozoite-only peak activity of mefloquine. Metabolically, lumefantrine induced minor peptide increases and clustered with GNF-Pf-5660, which is known to affect hemoglobin uptake without directly targeting hemozoin formation (Vanaerschot et al., 2017). The different stage specificity and metabolic profiles between lumefantrine and mefloquine suggest distinct mode of actions, despite PfMDR1 being a determinant of low-level resistance to both (Eastman and Fidock, 2009).

Methylene blue is known to act as a redox cycler and is used clinically to treat methemoglobinemia via its reduction of Fe^{3+} to Fe^{2+} (Blank et al., 2012). Methylene blue also binds hematin (a precursor of hemozoin crystals) at low micromolar concentrations *in vitro* (Blank et al., 2012). Our finding of similar stage specificity and metabolomic profiles between methylene blue and piperaquine suggest that both could affect heme detoxification and hemozoin formation, albeit via different mechanisms. Methylene blue potentially causes a reduction of Fe^{3+}, whereas piperaquine is presumed to bind Fe^{3+}-heme and prevent its incorporation into chemically inert hemozoin (Dhingra et al., 2017). Methylene blue, in contrast to piperaquine, is also potent against mature gametocytes that are not thought to degrade hemoglobin (Adjalley et al., 2011), implying an additional mode of action for methylene blue that might affect additional redox cycling agents such as NADPH levels (Siciliano et al., 2017).

Interestingly, ferroquine and naphthoquine, which are both chloroquine derivatives that are currently part of artemisinin-based combination therapies under clinical trials (Isba et al., 2015; Supan et al., 2017), shared a unique stage specificity profile showing peak activity during early rings and a gradual increase of IC_{50}^{8h} values through to schizonts (Figure 2). Ferroquine has hemozoin inhibitory activity similar to chloroquine and has been shown to induce the formation of hydroxyl radicals via the Fenton reaction, leading to lipid peroxidation and exacerbating oxidative stress in the parasite (Atamna and Ginsburg, 1993; Chavain et al., 2008; Dubar et al., 2008). This additional mode of action might contribute to the unique stage-specific profile of ferroquine action. The mode of action of naphthoquine is less understood. Even though parasites exposed to naphthoquine and ferroquine did not reveal major changes in the levels of detected metabolites thus causing them to cluster in the low fold change metabolic group (Figure 7), their shared and distinctive stage specificity profiles suggest a common target or pathway.

Compounds with peak activity during ring stages are highly desired. In our assays, naphthoquine, ferroquine, and WLL-vs showed peak activity specifically during ring stages, whereas chloroquine, piperaquine, methylene blue, dihydroartemisinin, lumefantrine, GNF-Pf-5660, MMV022224, and MMV668311 showed peak activity in rings and trophozoites. This diversity among ring-active compounds suggests the presence of multiple druggable processes in rings, despite this stage being considered less metabolically active (Allman et al., 2016) than trophozoites. One such process involves the proteasome, since the ring-active compound WLL-vs specifically binds to and inhibits the β2 and β5 subunits of the Pf 26S proteasome (Li et al., 2016; Stokes et al., 2019). Other processes that appear to begin early in rings include hemoglobin endocytosis and catabolism (Elliott et al., 2008; Vanaerschot et al., 2017; Xie et al., 2016).

We note that WLL-vs, included in our study, is a covalent binder of the Pf 26S proteasome inhibitor, meaning that wash-out protocols would have little effect on its irreversible mode of action. Previous studies on *Plasmodium* have shown that mRNA transcripts are produced in a "just-in-time" fashion, i.e., when they are needed for the parasite's development (Painter et al., 2018). This would suggest that the chances of falsely detecting early stage activity are minimal. However, a lingering effect after drug wash-out could theoretically result in overestimating compound activity during later stages. For this reason, we have included five different time points at which compound exposure was started, followed by drug wash-outs, to minimize compound carry over. This approach was validated with our WLL-vs data, which showed lower IC_{50}^{8h} values in rings compared with trophozoites and schizonts (Figure 2).

An established high-priority mode of action is inhibition of mitochondrial functions, targeting either DHODH (DSM265) or CytBC1 (atovaquone) (Goodman et al., 2017) (Figure 5A). Both, however, yield resistance at low inocula, which in patients translates into an increased risk of treatment failure using these classes of inhibitors (Llanos-Cuentas et al., 2018; Musset et al., 2007). The experimental compounds MMV000787, MMV021735, and MMV030666 showed peak activity in late trophozoites, albeit with incomplete killing, and shared the same distinct metabolic profile of increased dihydroorotate and N-carbamoyl-L-aspartate levels and decreased orotidine 5-P levels (Figure 7; Table S4) that is characteristic for DHODH and CytBC1 inhibition (Allman et al., 2016). Interestingly, selections with these former compounds failed to yield resistant parasites, even at high inocula of 5×10^8 parasites. They also did not show the biphasic curves observed for atovaquone and DSM265. These data raise the possibility that inhibition of mitochondrial pathways might be achievable through mode of actions that are distinct from DHODH and CytBC1 and that are less prone to acquisition of resistance. In addition to the metabolic signature of mitochondrial inhibition, MMV030666 also induced decreased peptide levels (Table S4), causing it to metabolically cluster with compounds inhibiting hemoglobin catabolism (Figure 7). However, MMV030666 still maintained a late trophozoite stage-specific activity profile similar to that of DSM265 and atovaquone but distinct from the overall trophozoite or ring plus trophozoite peak activity profiles usually observed for the majority of compounds with a hemoglobin catabolism metabolic signature (Figures 4, 5, and 7; Table S4). Cell morphological analysis of MMV030666-exposed parasites (Figure S4) identified late trophozoites as

the most sensitive intra-erythrocytic stage, consistent with mitochondrial inhibition, but also showed a bloated digestive vacuole that is characteristic for inhibitors of hemoglobin catabolism (Ross et al., 2018).

Most hits that clustered within the hemoglobin catabolism group, characterized by decreased peptide levels (Allman et al., 2016), showed peak activity in trophozoites (MMV027496, MMV019555, MMV000248, MMV006455, MMV007181, and MMV000442). The exceptions were GNF-Pf-5660 (Vanaerschot et al., 2017), lumefantrine, and MMV665794, which showed peak activity against rings and trophozoites. This observation, plus additional metabolic changes induced by MMV019555, highlights the potential diversity in mode of actions among compounds showing hemoglobin catabolism perturbation.

Among all compounds tested, MMV022224 was unique both in its metabolomic fingerprint and its stage specificity. Exposure to MMV022224 caused increased peptide levels and only this compound showed activity exclusively in rings and trophozoites but not in schizonts. Peptide accumulation may suggest a metabolic disruption further downstream in the hemoglobin catabolism pathway, possibly of an aminopeptidase or transporter. These unique profiles highlight MMV022224 as an attractive hit from a discovery and development perspective.

It is important to note that the metabolomics experiments in this study were exploratory in nature, involving one to two biological replicates to screen for known and novel candidate mode of actions within a large set of compounds. Once compounds are selected and prioritized for further discovery or development studies, these metabolomics data should be complemented with targeted in-depth follow-up studies to validate candidate targets and mode of actions as demonstrated recently for a new class of pantothenamides (Schalwijk et al., 2019).

The asexual blood stage susceptibility profiles of compounds may also help determine whether a protein is a target or solely a resistance mechanism. Resistance selections with MMV675939, MMV665939, and MMV020746 all identified SNPs or copy-number variations in the ABC transporter I family member 1, also known as ABCI3 (PF3D7_0319700) (Cowell et al., 2018). MMV675939 was most active on early and late trophozoites with IC_{50}^{8h} values that were only 2-fold higher than the IC_{50}^{72h} value, while MMV020746 and MMV665939 showed IC_{50}^{8h} values that were >28-fold higher than the IC_{50}^{72h} (Table S1). This contrast between the timing of peak activity for MMV675939 and the two other compounds suggests that they have different modes of actions and that ABCI3 is solely a resistance mediator and not the target.

Asexual blood stage susceptibility profiling may also help prioritize screening hits. Compounds with potent IC_{50}^{8h} values across all stages are of particular interest for further development as such activity profiles might compensate for a faster clearance or other pharmacokinetic-related issues that reduce *in vivo* exposure time. Dihydroartemisinin and piperaquine, two first-line antimalarial drugs, showed activity on all stages with IC_{50}^{8h} values at the most susceptible stages that were within 2-fold of their IC_{50}^{72h} values (Table S1). Chloroquine, mefloquine, and lumefantrine showed larger IC_{50}^{8h} over IC_{50}^{72h} ratios, but with IC_{50}^{8h} values still <300 nM (Table S1). Based on these parameters, ferroquine, WLL-vs, and GNF-PF-5660 represent promising antimalarial scaffolds. WLL-vs is of particular interest given its selectivity for the parasite proteasome and the fact that resistance is rare and low-grade (Li et al., 2016; Stokes et al., 2019; Yoo et al., 2018). Ferroquine has shown promising efficacy in phase II trials (Supan et al., 2017) and our assays indicated a unique ring-active profile that underscores its potential. With GNF-Pf-5660, chemical derivatization efforts are underway to improve its partial *in vivo* efficacy, established in rodent malaria models (Vanaerschot et al., 2017).

Compounds that show IC_{50}^{8h} values orders of magnitude larger than IC_{50}^{72h} values are potentially of less interest as these may have multiple mode of actions throughout intra-erythrocytic development and/or require longer exposures to achieve full killing. In addition, such a profile indicates that the short exposures usually applied for metabolomics will likely yield a less informative response. None of the current clinical or advanced candidate antimalarials showed this profile, suggesting that this is indeed a good de-prioritization criterion for further development. Examples of experimental compounds with such an unfavorable profile in our dataset were MMV022478 and MMV019017 (Figure 4), and MMV665939 and MMV020746, which showed IC_{50}^{8h} values >10 μM at all stages (data not shown) (Table S1).

MMV000442, MMV006455, MMV007181, and MMV665971 showed a peculiar profile in early and late ring stages, with initial growth inhibition at lower compound concentrations that reverses to less inhibition at higher concentrations (Figures 3 and 4). Solubility assays with MMV0004442, MMV006455, and MMV665791 indicated that these compounds have an aqueous solubility >100 μM, indicating that these survival curves are not due to solubility issues. This phenomenon has been observed in other chemical series and can at times be overcome through lead optimization (Le Manach et al., 2018). Despite their undesirable dose-response curves, these compounds might therefore still prove valuable as starting points for drug discovery efforts.

The asexual blood stage specificity profiles can also inform the selection of partner drugs for combination therapies. Ideally, combinations would target all different asexual blood stages. As an example, schizont-specific compounds could be partnered with compounds that target rings and trophozoites. These profiles can also be used to devise strategies to delay the emergence of resistance. For example, the late trophozoite-active compound DSM265 could be combined with another compound with a broader activity profile including late trophozoite to delay the emergence of DSM265 resistance (Llanos-Cuentas et al., 2018).

In summary, integrating investigations into antimalarial stage-specific mode of actions including metabolic perturbations into drug discovery and development programs should benefit ongoing efforts to develop new medicines to counter the spread of antimalarial multidrug resistance, as part of the mission to eliminate this disease.

SIGNIFICANCE

With the increasing spread of *Plasmodium falciparum* resistance to artemisinins and their partner drugs, the development of antimalarials with new modes of actions is more critical than ever. High-throughput screens are able to

identify potent chemical scaffolds, but not knowing their target often hampers their further development. Malaria drug discovery pipelines would thus greatly benefit from new assays that interrogate the mode of action and activity profile of screening hits. We designed an approach that provides more resolution into the different modes of action of clinical and experimental antimalarials by identifying the specific moment of asexual blood stage development against which these compounds are most active and combining this with a metabolomics assessment of pathway perturbations. This identified several stage specificity profiles that correlated well with inhibition of particular metabolic pathways. Interestingly, we also identified compounds that act on similar pathways albeit through different targets. Aside from generating insights into the tested clinical antimalarials, this approach also offered a rationale for the prioritization of experimental compounds. Our study identified several hits from the Malaria box and the Malaria Drug Accelerator consortium that showed promising antimalarial profiles for further development, especially in the context of combination therapies. Importantly, this approach can also be adopted for other pathogens that undergo multiple differentiation steps within their host.

STAR★METHODS

Detailed methods are provided in the online version of this paper and include the following:

- KEY RESOURCES TABLE
- LEAD CONTACT AND MATERIALS AVAILABILITY
- EXPERIMENTAL MODEL AND SUBJECT DETAILS
- METHOD DETAILS
  - Stage Specificity Assay
  - Culturing for Metabolomics
  - Metabolomics
  - Targeted Analysis
  - Resistance Selections
  - Solubility Assay
- QUANTIFICATION AND STATISTICAL ANALYSIS
- DATA AND CODE AVAILABILITY

SUPPLEMENTAL INFORMATION

Supplemental Information can be found online at https://doi.org/10.1016/j.chembiol.2019.11.009.

ACKNOWLEDGMENTS

We thank Tarrick Qahash for assistance with metabolomics data analysis. This work was supported by the Bill and Melinda Gates Foundation (OPP1054480), the Medicines for Malaria Venture (08/0015, to D.A.F.) and the NIH (R37 AI50234, R01 AI109023, R01 AI124678, and R33 AI127581).

AUTHOR CONTRIBUTIONS

J.M.M. and M.V. designed and performed stage specificity assays and analyzed the results. E.S.O. and M.L. designed metabolomic experiments, which were performed by E.S.O. and analyzed by E.S.O. and M.L. E.S.I. and M.C.S.L. performed resistance selections. S.O. sourced the compounds and provided input. J.M.M., M.V., E.S.O., M.L., and D.A.F. integrated the different datasets. M.V., D.A.F., E.A.W., D.E.G., and M.L. coordinated individual lab efforts. J.M.M., M.V., E.S.O., and D.A.F. wrote the manuscript, which was approved by all authors.

DECLARATION OF INTERESTS

The authors declare no competing interests.

Received: July 18, 2019
Revised: October 18, 2019
Accepted: November 14, 2019
Published: December 5, 2019

REFERENCES

Adjalley, S.H., Johnston, G.L., Li, T., Eastman, R.T., Ekland, E.H., Eappen, A.G., Richman, A., Sim, B.K., Lee, M.C., Hoffman, S.L., et al. (2011). Quantitative assessment of *Plasmodium falciparum* sexual development reveals potent transmission-blocking activity by methylene blue. Proc. Natl. Acad. Sci. U S A *108*, E1214–E1223.

Agrawal, S., Kumar, S., Sehgal, R., George, S., Gupta, R., Poddar, S., Jha, A., and Pathak, S. (2019). El-MAVEN: a fast, robust, and user-friendly mass spectrometry data processing engine for metabolomics. Methods Mol. Biol. *1978*, 301–321.

Allman, E.L., Painter, H.J., Samra, J., Carrasquilla, M., and Llinas, M. (2016). Metabolomic profiling of the malaria box reveals antimalarial target pathways. Antimicrob. Agents Chemother. *60*, 6635–6649.

Antonova-Koch, Y., Meister, S., Abraham, M., Luth, M.R., Ottilie, S., Lukens, A.K., Sakata-Kato, T., Vanaerschot, M., Owen, E., Jado, J.C., et al. (2018). Open-source discovery of chemical leads for next-generation chemoprotective antimalarials. Science *362*, https://doi.org/10.1126/science.aat9446.

Atamna, H., and Ginsburg, H. (1993). Origin of reactive oxygen species in erythrocytes infected with *Plasmodium falciparum*. Mol. Biochem. Parasitol. *61*, 231–241.

Beleites, C., and Sergo, V. (2018). Hyperspec: a package to handle hyperspectral data sets in R. R package version 0.99-20180627. http://hyperspec.r-forge.r-project.org/.

Blank, O., Davioud-Charvet, E., and Elhabiri, M. (2012). Interactions of the antimalarial drug methylene blue with methemoglobin and heme targets in *Plasmodium falciparum*: a physico-biochemical study. Antioxid. Redox Signal. *17*, 544–554.

Blasco, B., Leroy, D., and Fidock, D.A. (2017). Antimalarial drug resistance: linking *Plasmodium falciparum* parasite biology to the clinic. Nat. Med. *23*, 917–928.

Cassera, M.B., Zhang, Y., Hazleton, K.Z., and Schramm, V.L. (2011). Purine and pyrimidine pathways as targets in *Plasmodium falciparum*. Curr. Top. Med. Chem. *11*, 2103–2115.

Chavain, N., Vezin, H., Dive, D., Touati, N., Paul, J.F., Buisine, E., and Biot, C. (2008). Investigation of the redox behavior of ferroquine, a new antimalarial. Mol. Pharm. *5*, 710–716.

Chong, J., Soufan, O., Li, C., Caraus, I., Li, S., Bourque, G., Wishart, D.S., and Xia, J. (2018). MetaboAnalyst 4.0: towards more transparent and integrative metabolomics analysis. Nucleic Acids Res. *46*, W486–W494.

Combrinck, J.M., Mabotha, T.E., Ncokazi, K.K., Ambele, M.A., Taylor, D., Smith, P.J., Hoppe, H.C., and Egan, T.J. (2013). Insights into the role of heme in the mechanism of action of antimalarials. ACS Chem. Biol. *8*, 133–137.

Cowell, A.N., Istvan, E.S., Lukens, A.K., Gomez-Lorenzo, M.G., Vanaerschot, M., Sakata-Kato, T., Flannery, E.L., Magistrado, P., Owen, E., Abraham, M., et al. (2018). Mapping the malaria parasite druggable genome by using *in vitro* evolution and chemogenomics. Science *359*, 191–199.

Delves, M.J., Miguel-Blanco, C., Matthews, H., Molina, I., Ruecker, A., Yahiya, S., Straschil, U., Abraham, M., Leon, M.L., Fischer, O.J., et al. (2018). A high throughput screen for next-generation leads targeting malaria parasite transmission. Nat. Commun. *9*, 3805.

Dhingra, S.K., Redhi, D., Combrinck, J.M., Yeo, T., Okombo, J., Henrich, P.P., Cowell, A.N., Gupta, P., Stegman, M.L., Hoke, J.M., et al. (2017). A variant

PfCRT isoform can contribute to *Plasmodium falciparum* resistance to the first-line partner drug piperaquine. MBio *8*, e00303-17.

Dubar, F., Khalife, J., Brocard, J., Dive, D., and Biot, C. (2008). Ferroquine, an ingenious antimalarial drug: thoughts on the mechanism of action. Molecules *13*, 2900–2907.

Duffy, S., and Avery, V.M. (2017). *Plasmodium falciparum in vitro* continuous culture conditions: a comparison of parasite susceptibility and tolerance to anti-malarial drugs throughout the asexual intra-erythrocytic life cycle. Int. J. Parasitol. Drugs Drug Resist. *7*, 295–302.

Eastman, R.T., and Fidock, D.A. (2009). Artemisinin-based combination therapies: a vital tool in efforts to eliminate malaria. Nat. Rev. Microbiol. *7*, 864–874.

Ekland, E.H., Schneider, J., and Fidock, D.A. (2011). Identifying apicoplast-targeting antimalarials using high-throughput compatible approaches. FASEB J. *25*, 3583–3593.

Elliott, D.A., McIntosh, M.T., Hosgood, H.D., 3rd, Chen, S., Zhang, G., Baevova, P., and Joiner, K.A. (2008). Four distinct pathways of hemoglobin uptake in the malaria parasite *Plasmodium falciparum*. Proc. Natl. Acad. Sci. U S A *105*, 2463–2468.

Fang, H., and Gough, J. (2014). SupraHex: an R/Bioconductor package for tabular omics data analysis using a supra-hexagonal map. Biochem. Biophys. Res. Commun. *443*, 285–289.

Gamo, F.J., Sanz, L.M., Vidal, J., de Cozar, C., Alvarez, E., Lavandera, J.L., Vanderwall, D.E., Green, D.V., Kumar, V., Hasan, S., et al. (2010). Thousands of chemical starting points for antimalarial lead identification. Nature *465*, 305–310.

Gomez-Lorenzo, M.G., Rodriguez-Alejandre, A., Moliner-Cubel, S., Martinez-Hoyos, M., Bahamontes-Rosa, N., Gonzalez Del Rio, R., Rodenas, C., Fuente, J., Lavandera, J.L., Garcia-Bustos, J.F., et al. (2018). Functional screening of selective mitochondrial inhibitors of *Plasmodium*. Int. J. Parasitol. Drugs Drug Resist. *8*, 295–303.

Goodman, C.D., Buchanan, H.D., and McFadden, G.I. (2017). Is the mitochondrion a good malaria drug target? Trends Parasitol. *33*, 185–193.

Guiguemde, W.A., Shelat, A.A., Bouck, D., Duffy, S., Crowther, G.J., Davis, P.H., Smithson, D.C., Connelly, M., Clark, J., Zhu, F., et al. (2010). Chemical genetics of *Plasmodium falciparum*. Nature *465*, 311–315.

Isba, R., Zani, B., Gathu, M., and Sinclair, D. (2015). Artemisinin-naphthoquine for treating uncomplicated *Plasmodium falciparum* malaria. Cochrane Database Syst. Rev. CD011547. https://doi.org/10.1002/14651858.CD011547.

Jomaa, H., Wiesner, J., Sanderbrand, S., Altincicek, B., Weidemeyer, C., Hintz, M., Turbachova, I., Eberl, M., Zeidler, J., Lichtenthaler, H.K., et al. (1999). Inhibitors of the nonmevalonate pathway of isoprenoid biosynthesis as antimalarial drugs. Science *285*, 1573–1576.

Le Manach, C., Paquet, T., Wicht, K., Nchinda, A.T., Brunschwig, C., Njoroge, M., Gibhard, L., Taylor, D., Lawrence, N., Wittlin, S., et al. (2018). Antimalarial lead-optimization studies on a 2,6-imidazopyridine series within a constrained chemical space to circumvent atypical dose-response curves against multidrug resistant parasite strains. J. Med. Chem. *61*, 9371–9385.

Li, H., O'Donoghue, A.J., van der Linden, W.A., Xie, S.C., Yoo, E., Foe, I.T., Tilley, L., Craik, C.S., da Fonseca, P.C., and Bogyo, M. (2016). Structure- and function-based design of *Plasmodium*-selective proteasome inhibitors. Nature *530*, 233–236.

Linares, M., Viera, S., Crespo, B., Franco, V., Gomez-Lorenzo, M.G., Jimenez-Diaz, M.B., Angulo-Barturen, I., Sanz, L.M., and Gamo, F.J. (2015). Identifying rapidly parasiticidal anti-malarial drugs using a simple and reliable *in vitro* parasite viability fast assay. Malar. J. *14*, 441.

Llanos-Cuentas, A., Casapia, M., Chuquiyauri, R., Hinojosa, J.C., Kerr, N., Rosario, M., Toovey, S., Arch, R.H., Phillips, M.A., Rozenberg, F.D., et al. (2018). Antimalarial activity of single-dose DSM265, a novel *Plasmodium* dihydroorotate dehydrogenase inhibitor, in patients with uncomplicated *Plasmodium falciparum* or *Plasmodium vivax* malaria infection: a proof-of-concept, open-label, phase 2a study. Lancet Infect. Dis. *18*, 874–883.

McNamara, C.W., Lee, M.C., Lim, C.S., Lim, S.H., Roland, J., Simon, O., Yeung, B.K., Chatterjee, A.K., McCormack, S.L., Manary, M.J., et al. (2013). Targeting *Plasmodium* PI(4)K to eliminate malaria. Nature *504*, 248–253.

Menard, D., and Dondorp, A. (2017). Antimalarial drug resistance: a threat to malaria elimination. Cold Spring Harb. Perspect. Med. *7*, a025619.

Miller, L.H., Baruch, D.I., Marsh, K., and Doumbo, O.K. (2002). The pathogenic basis of malaria. Nature *415*, 673–679.

Musset, L., Le Bras, J., and Clain, J. (2007). Parallel evolution of adaptive mutations in *Plasmodium falciparum* mitochondrial DNA during atovaquone-proguanil treatment. Mol. Biol. Evol. *24*, 1582–1585.

Okombo, J., and Chibale, K. (2017). Insights into integrated lead generation and target identification in malaria and tuberculosis drug discovery. Acc. Chem. Res. *50*, 1606–1616.

Paguio, M.F., Bogle, K.L., and Roepe, P.D. (2011). *Plasmodium falciparum* resistance to cytocidal versus cytostatic effects of chloroquine. Mol. Biochem. Parasitol. *178*, 1–6.

Painter, H.J., Chung, N.C., Sebastian, A., Albert, I., Storey, J.D., and Llinas, M. (2018). Genome-wide real-time *in vivo* transcriptional dynamics during *Plasmodium falciparum* blood-stage development. Nat. Commun. *9*, 2656.

Painter, H.J., Morrisey, J.M., and Vaidya, A.B. (2010). Mitochondrial electron transport inhibition and viability of intraerythrocytic *Plasmodium falciparum*. Antimicrob. Agents Chemother. *54*, 5281–5287.

Phillips, M.A., Lotharius, J., Marsh, K., White, J., Dayan, A., White, K.L., Njoroge, J.W., El Mazouni, F., Lao, Y., Kokkonda, S., et al. (2015). A long-duration dihydroorotate dehydrogenase inhibitor (DSM265) for prevention and treatment of malaria. Sci. Transl. Med. *7*, 296ra111.

Phillips, M.A., Burrows, J.N., Manyando, C., van Huijsduijnen, R.H., Van Voorhis, W.C., and Wells, T.N.C. (2017). Malaria. Nat. Rev. Dis. Primers *3*, 17050.

Plouffe, D., Brinker, A., McNamara, C., Henson, K., Kato, N., Kuhen, K., Nagle, A., Adrian, F., Matzen, J.T., Anderson, P., et al. (2008). *In silico* activity profiling reveals the mechanism of action of antimalarials discovered in a high-throughput screen. Proc. Natl. Acad. Sci. U S A *105*, 9059–9064.

Raphemot, R., Lafuente-Monasterio, M.J., Gamo-Benito, F.J., Clardy, J., and Derbyshire, E.R. (2015). Discovery of dual-stage malaria inhibitors with new targets. Antimicrob. Agents Chemother. *60*, 1430–1437.

Ross, L.S., and Fidock, D.A. (2019). Elucidating mechanisms of drug-resistant *Plasmodium falciparum*. Cell Host Microbe *26*, 35–47.

Ross, L.S., Dhingra, S.K., Mok, S., Yeo, T., Wicht, K.J., Kumpornsin, K., Takala-Harrison, S., Witkowski, B., Fairhurst, R.M., Ariey, F., et al. (2018). Emerging Southeast Asian PfCRT mutations confer *Plasmodium falciparum* resistance to the first-line antimalarial piperaquine. Nat. Commun. *9*, 3314.

RStudio Team (2015). RStudio: integrated development for R. RStudio, Inc., Boston, MA. http://www.rstudio.com/.

Sanz, L.M., Crespo, B., De-Cozar, C., Ding, X.C., Llergo, J.L., Burrows, J.N., Garcia-Bustos, J.F., and Gamo, F.J. (2012). *P. falciparum in vitro* killing rates allow to discriminate between different antimalarial mode-of-action. PLoS One *7*, e30949.

Schalwijk, J., Allman, E.L., Jansen, P.A.M., de Vries, L.E., Verhoef, J.M.J., Jackowski, S., Botman, P.N.M., Beuckens-Schortinghuis, C.A., Koolen, K.M.J., Bolscher, J.M., et al. (2019). Antimalarial pantothenamide metabolites target acetyl-coenzyme A biosynthesis in *Plasmodium falciparum*. Sci. Transl. Med. *11*, eaaas9917.

Siciliano, G., Santha Kumar, T.R., Bona, R., Camarda, G., Calabretta, M.M., Cevenini, L., Davioud-Charvet, E., Becker, K., Cara, A., Fidock, D.A., et al. (2017). A high susceptibility to redox imbalance of the transmissible stages of *Plasmodium falciparum* revealed with a luciferase-based mature gametocyte assay. Mol. Microbiol. *104*, 306–318.

Stokes, B.H., Yoo, E., Murithi, J.M., Luth, M.R., Afanasyev, P., da Fonseca, P.C.A., Winzeler, E.A., Ng, C.L., Bogyo, M., and Fidock, D.A. (2019). Covalent *Plasmodium falciparum*-selective proteasome inhibitors exhibit a low propensity for generating resistance *in vitro* and synergize with multiple antimalarial agents. PLoS Pathog. *15*, e1007722.

Supan, C., Mombo-Ngoma, G., Kombila, M., Ospina Salazar, C.L., Held, J., Lell, B., Cantalloube, C., Djeriou, E., Ogutu, B., Waitumbi, J., et al. (2017). Phase 2a, open-label, 4-escalating-dose, randomized multicenter study evaluating the safety and activity of ferroquine (SSR97193) plus artesunate, versus amodiaquine plus artesunate, in African adult men with uncomplicated *Plasmodium falciparum* malaria. Am. J. Trop. Med. Hyg. *97*, 514–525.

Vanaerschot, M., Lucantoni, L., Li, T., Combrinck, J.M., Ruecker, A., Kumar, T.R.S., Rubiano, K., Ferreira, P.E., Siciliano, G., Gulati, S., et al. (2017). Hexahydroquinolines are antimalarial candidates with potent blood-stage and transmission-blocking activity. Nat. Microbiol. *2*, 1403–1414.

Veiga, M.I., Dhingra, S.K., Henrich, P.P., Straimer, J., Gnadig, N., Uhlemann, A.C., Martin, R.E., Lehane, A.M., and Fidock, D.A. (2016). Globally prevalent PfMDR1 mutations modulate *Plasmodium falciparum* susceptibility to artemisinin-based combination therapies. Nat. Commun. *7*, 11553.

WHO (2018). World Malaria Report 2018 (World Health Organization).

Wilson, D.W., Langer, C., Goodman, C.D., McFadden, G.I., and Beeson, J.G. (2013). Defining the timing of action of antimalarial drugs against *Plasmodium falciparum*. Antimicrob. Agents Chemother. *57*, 1455–1467.

Wu, W., Herrera, Z., Ebert, D., Baska, K., Cho, S.H., DeRisi, J.L., and Yeh, E. (2015). A chemical rescue screen identifies a *Plasmodium falciparum* apicoplast inhibitor targeting MEP isoprenoid precursor biosynthesis. Antimicrob. Agents Chemother. *59*, 356–364.

Xie, S.C., Dogovski, C., Hanssen, E., Chiu, F., Yang, T., Crespo, M.P., Stafford, C., Batinovic, S., Teguh, S., Charman, S., et al. (2016). Haemoglobin degradation underpins the sensitivity of early ring stage *Plasmodium falciparum* to artemisinins. J. Cell Sci. *129*, 406–416.

Yoo, E., Stokes, B.H., de Jong, H., Vanaerschot, M., Kumar, T., Lawrence, N., Njoroge, M., Garcia, A., Van der Westhuyzen, R., Momper, J.D., et al. (2018). Defining the determinants of specificity of *Plasmodium* proteasome inhibitors. J. Am. Chem. Soc. *140*, 11424–11437.

Zhang, Y., Asante, K.S., and Jung, A. (1986). Stage-dependent inhibition of chloroquine on *Plasmodium falciparum in vitro*. J. Parasitol. *72*, 830–836.

STAR★METHODS

KEY RESOURCES TABLE

REAGENT or RESOURCE	SOURCE	IDENTIFIER
Chemicals, Peptides, and Recombinant Proteins		
All tested antimalarials and their structures are available in Table S2 and Figure S2.		
Experimental Models: Cell Lines		
Parasite line 3D7-A10	Goldberg lab at Washington State Univesity, St. Louis, USA	3D7-A10
Parasite line 3D7-MR4	Malaria Research and Reference Reagent Resource Center	Cat#MRA-104
Parasite line Dd2-B2	Wellems Lab at NIAID, MD, USA	Dd2-B2
Other		
All tested antimalarials and their structures are available in Table S2 and Figure S2.		
Software and Algorithms		
GraphPad Prism 8	GraphPad Software, San Diego, CA, USA	www.graphpad.com
El-MAVEN	Agrawal et al., 2019	https://elucidatainc.github.io/ElMaven/
RStudio	RStudio Team, 2015	http://www.rstudio.com/
Metaboanalyst	Chong et al., 2018	https://www.metaboanalyst.ca/
Hyperspec	Beleites and Sergo, 2018.	http://hyperspec.r-forge.r-project.org
Suprahex R	Fang and Gough, 2014	http://supfam.org/supraHex

LEAD CONTACT AND MATERIALS AVAILABILITY

Further information and requests for resources and reagents should be directed to and will be fulfilled by the Lead Contact, Manu Vanaerschot (manu.vanaerschot@gmail.com). Please note that availability of experimental compounds may be restricted and might require resynthesis. All chemical structures as well as SMILES for each compound are available in Figures S1 and S2 and Table S2.

EXPERIMENTAL MODEL AND SUBJECT DETAILS

The Pf parasites used in this study were cultured in human O^+ blood (sex of donor unknown) at 3% hematocrit in RPMI-1640 media supplemented with 50 μM hypoxanthine, 2 g L^{-1} sodium bicarbonate, 2 mM L-glutamine, 25 mM HEPES, 0.5% AlbuMAXII (Invitrogen) and 10 μg mL^{-1} gentamycin in 5% O_2, 5% CO_2 and 90% N_2 at 37°C. The 3D7-A10 Pf line is a clone of the 3D7 line received from the Goldberg lab at Washington State University in St. Louis. The 3D7-MR4 line was obtained from the Malaria Research and Reference Reagent Resource Center (MR4, Cat#MRA-102). The Dd2-B2 Pf line is a clone obtained by limited dilution from the Dd2 line provided by Dr. Thomas Wellems (NIAID, NIH).

METHOD DETAILS

Stage Specificity Assay

Standard asexual blood stage susceptibility results were collected by exposing asynchronous 3D7-A10 parasite cultures to 10 different concentrations plus no-compound controls for 72 hr. To determine the specific asexual blood stage at which the compounds are active, schizonts were magnetically purified using MACS LD columns (Miltenyi Biotec) from cultures that had been repeatedly synchronized with 5% sorbitol. After a 3hr incubation at 2% hematocrit to allow re-invasion, cultures were again sorbitol-synchronized to obtain a pure ring-stage culture (time = 0 hr). These parasites were then plated in five 96-well plates and exposed to compounds (SMILES and origin listed in Table S2, structures shown in Figures S1 and S2) as early rings (0-8 hr), late rings (8-16 hr), early trophozoites (16-24 hr), late trophozoites (24-32 hr) or schizonts (32-40 hr). Incubation times were adjusted to the 40 hr asexual blood stage cycle of the 3D7-A10 parasite line. Synchronicity of the cultures was confirmed by imaging on average 83 parasites per time point in control conditions. Compounds were removed through three rounds of washing including two plate changes in 37°C

prewarmed culture media after each exposure. All pipetting steps to expose and wash parasites were performed using a Tecan Freedom Evo 100 for increased throughput and accuracy. Each group of plates per timepoint were placed in a separate humidified chamber to avoid any delay in growth rate due to temperature variations. For the stage specificity assay, growth inhibition was assessed at the 60 hr time point at which parasites had expanded, reinvaded new RBCs, and developed into the trophozoite stage that allows straight-forward quantification by flow cytometry. This is very similar to the standard 72 hr assay in which parasites are not synchronized, but also allowed to reinvade and develop further for another half life cycle. Parasite survival for both the 72 hr and stage-specific 8 hr exposures was assessed by SYBR Green and MitoTracker Deep Red FM staining (Life Technologies) and subsequent flow-cytometric analysis (Accuri C6, BD Biosciences) (Ekland et al., 2011). IC_{50} values were derived from growth inhibition data using nonlinear regression (Prism 7, GraphPad). All asexual blood stage assays were repeated on at least three independent occasions with two technical replicates.

Culturing for Metabolomics

3D7-MR4 parasites were cultured at 50 ml volumes and 2% hematocrit as described elsewhere (Allman et al., 2016). Cultures were kept at the appropriate temperature and gas mixture in incubators between media exchange, culture division, and synchronization. Synchronization was achieved via 5% sorbitol. All reagents and experimental spaces were mycoplasma-free, and reagents passed through 0.2 μm liquid filters when possible prior to use.

Metabolomics

Hydrophilic metabolite changes in response to compound exposure were profiled as previously described (Allman et al., 2016). Treatments were performed on 1×10^8 MACS-purified, synchronized trophozoite parasite-infected RBCs (24-36 hr post invasion) in 5 mL RPMI. Compounds were added at a concentration of $10 \times IC_{50}^{72hr}$ and incubated for 2.5 hr. All treatment conditions were performed as technical triplicates and included an untreated control. Subsequently, PBS washes were performed, and infected RBCs were extracted with 90% methanol containing 0.5 μM $^{13}C^{15}N$-labelled aspartate as an internal standard, then dried under nitrogen and stored at -80°C. Process blanks were generated at the time of extraction in technical triplicates. Samples were then resuspended in high-performance liquid chromatography (HPLC) grade water containing 1 μM chlorpropamide as an additional internal standard and analyzed by ultra-high-performance liquid chromatography mass spectrometry UHPLC-MS as described (Allman et al., 2016).

Targeted Analysis

Following negative ionization analysis of hydrophilic extracts on a Thermo Exactive Plus Orbitrap, sample data were converted and transferred for analysis. Targeted peak picking from a curated list of 298 metabolites was achieved using el-MAVEN software (https://elucidatainc.github.io/ElMaven/ (Agrawal et al., 2019)), followed by normalization and analysis via RStudio (http://www.rstudio.com/) and Metaboanalyst (https://www.metaboanalyst.ca/ (Chong et al., 2018)). Data were visualized using the Hyperspec (http://hyperspec.r-forge.r-project.org) and Suprahex R (Fang and Gough, 2014) scripting packages in RStudio. Hierarchical clustering of the metabolic profiles to identify related metabolic signatures was performed using the Ward method, based on the Pearson correlation coefficients, by the Hyperspec R integrated heatmap function.

Resistance Selections

Attempts to obtain parasites resistant to MMV030666, MMV000787 and MMV021735 were performed using either single step (continuous) or ramping selection protocols as described elsewhere (Cowell et al., 2018). For single step selections, parasites are continuously exposed to relatively high concentrations of the compound of interest (usually $3 \times IC_{50}$) with culture media and RBCs being regularly refreshed until actively growing parasites are again observed. Cultures were monitored for minimum 70 days after start of exposure. For ramping selections, parasites are exposed at low compound concentrations (usually $1 \times IC_{50}$ or lower) and parasite growth is continuously monitored. When parasites seem to have adapted to the pressure, compound concentrations are gradually increased to adapt parasites to even higher levels of compound. Standard IC_{50}^{72hr} assays were performed on recrudesced parasites from single step selections, if any, and on parasites resulting from ramping selections.

Solubility Assay

The aqueous solubility of MMV007181, MMV000442 and MMV006455 was determined at a single concentration of 500 μM because of compound scarcity. The protocol used was adapted from Millipore Corporation's "MultiScreen® Solubility Filter Plate" application note. Dihydroartemisinin, chloroquine and piperaquine were used as controls. Briefly, compounds were first dissolved in DMSO at 10 mM. They were then added to 1 × PBS (pH 7.4) at a 1:20 ratio in 1.5 ml tubes and mixed on a shaker (100 rpm) at room temperature for 1.5 hr. They were then filtered using Target2 regenerated cellulose 0.2 μM filters (Thermal Scientific, part number F2500-8) to remove any precipitate. 160 μl of the filtrate was dispensed into flat-bottomed 96-well culture plates and diluted with 40 μl/well acetonitrile. The plate was then placed on a shaker (100 rpm) at room temperature for 10 min. After mixing, the filtrate was analyzed using a Spectramax 340PC (Molecular Devices) at 280, 300, 320, 340, 360 and 800 nm. Standards were made by adding compounds into standards buffer (80:20 1 × PBS: acetonitrile, pH 7.4) at a 1:25 ratio. The mixtures were allowed to mix on a shaker (100 rpm) for

10 min at room temperature and analyzed at the same six wavelengths as mentioned above. The aqueous solubility of compounds was then determined by calculating the ratio of absorbances between the filtrate and the standard using the formula below:

$$\frac{(\sum \text{AU at } 280,\ 300,\ 320,\ 340,\ 360\ \text{nm}) - (\text{AU at } 800\ \text{nm})\ \text{Filtrate}}{(\sum \text{AU at } 280,\ 300,\ 320,\ 340,\ 360\ \text{nm}) - (\text{AU at } 800\ \text{nm})\ \text{Standard}}$$

If the ratio is ≈ 1, a compound's aqueous solubility is $\geq 500\ \mu M$. Ratios < 1.0 and > 0.5 indicate a solubility between 100 μM and 500 μM, while ratios ≤ 0.5 indicate a solubility $\leq 100\ \mu M$.

QUANTIFICATION AND STATISTICAL ANALYSIS

All details of the stage specificity experiments, including the number of biological (n), can be found in Table S1. The standard error of the mean (SEM) was used to report error values for means based on multiple independent repeats. Details on the number of repeats of the metabolomics study can be found in the legend of Table S4. This study did not perform any other statistical methods on the data.

DATA AND CODE AVAILABILITY

The published article includes all datasets generated in this study. The IC_{50} data and survival curves are available in Table S1 and Figures 1, 2, 3, 4, and 5. Metabolomics data is available in Table S4.

Cell Chemical Biology, Volume *27*

Supplemental Information

Combining Stage Specificity and Metabolomic Profiling to Advance Antimalarial Drug Discovery

James M. Murithi, Edward S. Owen, Eva S. Istvan, Marcus C.S. Lee, Sabine Ottilie, Kelly Chibale, Daniel E. Goldberg, Elizabeth A. Winzeler, Manuel Llinás, David A. Fidock, and Manu Vanaerschot

Table S1 (related to Figures 1–6). Asexual blood stage-specific IC_{50}^{8h} data in nM for the tested antimalarials.

Compound	Early rings			Late rings			Early trophozoites			Late trophozoites			Schizonts			Overall (72hr assay)			Peak Activity	Ratio lowest $IC_{50}^{8hr}/IC_{50}^{72hr}$
	Mean IC_{50}	SEM	n	Mean IC_{50}	SEM	n	Mean IC_{50}	SEM	n	Mean IC_{50}	SEM	n	Mean IC_{50}	SEM	n	Mean IC_{50}	SEM	n		
Dihydroartemisinin	1.5	0.1	3	2.4	0.1	3	2.4	0.4	3	3.9	0.2	3	11.6	1.3	3	1.0	0.1	4	Rings + Trophs	1.5
Chloroquine	17.8	5.5	4	18.6	2.5	3	17.4	3.5	3	30.1	6.3	3	60.3	13.4	3	5.9	0.8	3	Rings + Trophs	2.9
Mefloquine	258	106	3	147	74	3	28.8	7.0	3	29.5	6.9	3	138	39	3	9.8	1.0	3	Trophs	3.0
Lumefantrine	15.4	4.0	3	15.8	3.5	3	19.54	1.6	3	22.3	2.0	3	81.0	32.5	3	1.3	0.0	3	Rings + Trophs	11.6
Piperaquine	26.2	1.1	3	46.3	13.1	3	27.4	3.3	3	32.9	1.1	3	67.0	9.6	3	15.4	2.3	4	Rings + Trophs	1.7
Ferroquine	4.9	0.3	3	10.9	1.7	3	23.2	2.4	3	36.8	3.3	3	49.3	6.2	3	3.5	0.4	4	Rings	1.4
Methylene blue	3.2	0.3	3	13.3	2.7	3	5.9	0.4	3	8.3	2.0	3	64.8	17.8	3	1.8	0.1	4	Rings + Trophs	1.8
KAI407 - shift 1	-	-	-	-	-	-	-	-	-	161	44.0	3	-	-	-	-	-	-	-	3.9
KAI407 - shift 2	16263	904	2	14021	923	3	5139	1727	3	13386	354	2	46.2	0.6	3	11.8	1.0	3	Schizonts	-
AN3661	> 1.25 µM	-	3	> 1.25 µM	-	3	> 1.25 µM	-	3	138	39	3	400	34	3	35.6	3.6	3	Late Trophs	3.9
KAE609	> 1.25 µM	-	3	> 1.25 µM	-	3	3.2	0.6	3	1.1	0.2	3	1.2	0.1	3	0.73	0.05	4	Trophs + Schizonts	1.5
WLL-vs	23.7	2.6	3	29.4	4.4	3	57.6	3.1	3	47.1	3.1	3	50.1	5.7	3	7.2	0.8	3	Rings	3.3
DSM265 - shift 1	-	-	-	-	-	-	10.2	2.2	3	7.7	0.9	3	19.2	2.3	3	3.8	0.1	3	Late Trophs	2.0
DSM265 - shift 2	4821	1605	3	1493	308	3	1481	240	3	-	-	-	> 25 µM	-	-	-	-	-	-	-
Atovaquone - shift 1	-	-	-	-	-	-	0.68	0.06	3	3.7	0.2	3	7.4	1.3	3	1.1	0.1	3	Late Trophs	3.3
Atovaquone - shift 2	553	228	3	324	45	3	619	132	3	-	-	3	> 25 µM	-	3	-	-	-	-	-
Fosmidomycin	> 10 µM	-	3	> 10 µM	-	3	1983	665	3	1993	117	3	> 10 µM	-	3	405	8	3	Trophs	4.9
GNF-Pf-5660	119	30	3	238	57	3	108	6	3	79.3	10.8	3	290	50	3	4.4	0.2	3	Rings + Trophs	18.0
MMV665794	369	101	3	590	177	3	210	20	3	165	10	3	203	8	3	91.3	5.2	4	Trophs + Schizonts	1.8
Naphtoquine	4.2	1.8	3	8.1	0.4	3	19.5	2.9	3	29.2	3.1	3	42.8	8.4	3	3.2	0.4	4	Rings	1.3
MMV000442	> 8 µM	-	3	> 8 µM	-	3	176	11	3	197	22	3	503	48	3	37.2	3.1	3	Trophs	4.7
MMV675939	1170	277	3	268	84	3	62.9	29.9	3	146	54	3	1647	45	3	30.8	4.4	5	Trophs	2.0
MMV085071	893	78	3	492	122	3	61.1	0.1	3	81.4	2.8	3	254	47	3	105	11	3	Trophs	0.6
MMV668311	140	39	3	97.7	4.3	3	68.9	11.9	3	122	26	3	382	79	3	63.5	1.7	3	Rings + Trophs	1.1
MMV020746	> 25 µM	-	2	> 25 µM	-	2	12587	1681	2	> 25 µM	-	2	> 25 µM	-	2	56.8	6.6	4	All stages > 10 µM	221.5
MMV667491	2264	349	3	1270	92	3	521	28	3	731	50	3	842	108	3	243	35	4	Trophs + Schizonts	2.1
MMV006455	> 12.5 µM	-	3	> 12.5 µM	-	3	441	10	3	450	46	3	933	142	3	439	46	4	Trophs	1.0
MMV022478	7628	202	2	4870	353	2	2132	252	2	2529	353	2	3370	269	2	11.1	0.8	3	Trophs + Schizonts	192.4
MMV007181	> 25 µM	-	3	> 25 µM	-	3	253	27	3	274	16	3	570	107	3	84.9	8.9	3	Trophs	3.0
MMV665971	> 25 µM	-	3	> 25 µM	-	3	496	51	3	485	26	3	523	29	3	185	9	3	Trophs + Schizonts	2.6
MMV665939	17011	2420	3	22853	5330	3	13476	4752	3	39017	13066	3	54384	14265	3	478	49	4	All stages > 10 µM	28.2
MMV019017	8418	1862	2	8663	2298	3	1338	133	3	1708	116	3	3060	229	3	306	12	3	Trophs + Schizonts	4.4
MMV000248	n.d.	n.d.	0	6744	-	1	411	-	1	376	-	1	754	-	1	103	14	4	Trophs	3.7
MMV021735	> 25 µM	-	3	> 25 µM	-	3	5422	1392	3	234	12	3	5281	622	3	232	11	4	Late Trophs	1.0
MMV022224	838	123	3	845	152	3	542	39	3	752	58	3	2486	472	3	199	31	5	Rings + Trophs	2.7
MMV027496	2760	529	3	878	130	3	168	26	3	199	9	3	4339	545	3	89.7	7.9	3	Trophs	1.9
MMV019555	755	159	3	788	221	3	81.4	1.6	3	110	20	3	349	66	3	33.3	1.8	3	Trophs	2.4
MMV030666	10264	3053	3	11440	861	3	5124	506	3	858	29	3	4191	307	3	462	18	4	Late Trophs	1.9
MMV000787	5737	1215	3	7870	731	3	3312	629	3	1354	57	3	5912	617	3	1960	83	3	Late Trophs	0.7

Shift 1 and shift 2 indicate the two half-maximal inhibitory concentrations for biphasic dose response curves. SEM: standard error of the mean; n: number of biological repeats; - : no data; IC_{50}^{8h}: IC_{50} based on 8-h exposure; IC_{50}^{72h}: IC_{50} based on 72-h exposure.

Table S2 (related to Figures 1–6). SMILES and suspected mode of action (if known) of the tested antimalarials.

Compound	SMILES	Suspected mode of action	Origin candidate antimalarials	Alternative name
Dihydroartemisinin (DHA)	C[C@@H]1CC[C@H]2[C@H]([C@H](O[C@H]3[C@@]24[C@H]1CCC(O3)(OO4)C)O)C	-	-	-
Chloroquine (CQ)	CCN(CC)CCCC(C)NC1=C2C=CC(=CC2=NC=C1)Cl	β-hematin[a]	-	-
Mefloquine (MFQ)	c1cc2c(cc(nc2c(c1)C(F)(F)F)C(F)(F)F)[C@@H]([C@H]3CCCCN3)O	-	-	-
Lumefantrine (LMF)	CCCCN(CCCC)CC(C1=C2C3=C(C=C(C=C3)Cl)C(=CC4=CC=C(C=C4)Cl)C2=CC(=C1)Cl)O	-	-	-
Piperaquine (PPQ)	c1cc2c(ccnc2cc1Cl)N3CCN(CC3)CCCN4CCN(CC4)c5ccnc6c5ccc(c6)Cl	β-hematin[b]	-	-
Ferroquine (FQ)	CN(C)CC1=C(C=[C-]C1)CNC2=C3C=CC(=CC3=NC=C2)Cl.C1C=CC=[C-]1.[Fe+2]	β-hematin[c,d]	-	-
Methylene blue (MB)	CN(C)C1=CC2=C(C=C1)N=C3C=CC(=[N+](C)C)C=C3S2.[Cl-]	β-hematin[e]	-	-
KAI407	O=C(N(C)C1=CC=C(C=C1)C#N)C2=CN3C(C=N2)=NC=C3C4=CC=C(C=C4)C(F)(F)F	PI4K[f]	-	-
AN3661	OB1C2=C(CCC(O)=O)C=CC=C2CO1	CPSF[g]	-	-
KAE609	ClC1=C(F)C=C2C(NC3=C2C[C@H](C)N[C@@]34C(CC5=C4C=C(Cl)C=C5)=O)=C1	ATP4[h]	-	-
WLL-vs	CC(C)C[C@@H](/C=C/S(=O)(=O)C)NC(=O)[C@H](CC(C)C)NC(=O)[C@H](CC1=CNC2=CC=CC=C21)NC(=O)CN3CCOCC3	Proteasome[i,j]	-	-
DSM265	FS(F)(F)(F)(C1=CC=C(NC2=CC(C)=NC3=NC(C(F)(F)C)=NN23)C=C1)F	DHODH[k]	-	-
Atovaquone (ATQ)	O=C1C([C@@H]2CC[C@@H](C3=CC=C(Cl)C=C3)CC2)=C(O)C(C4=CC=CC=C41)=O	CYTB[l]	-	-
Fosmidomycin	C(CN(C=O)O)CP(=O)(O)O	DXR[m]	-	-
GNF-Pf-5660	CCOC(=O)C1=C(C)NC2=C(C1C3=CC=CC=C3Cl)C(=O)CC(C2)C4=CC=C(OC)C(OC)=C4	*[n]	-	-
Naphthoquine (NQ)	CC(C)(C)NCc1cc(c2c(c1O)CCCC2)Nc3ccnc4c3ccc(c4)Cl	-	-	MMV000017
MMV665794	FC(F)(F)C1=CC(NC2=C(NC3=CC(=CC=C3)C(F)(F)F)N=C3C=CC=CC3=N2)=CC=C1	-	MMV Malaria Box	-
MMV000442	CC(C)(C)c1ccc2OCN(Cc3ccc(Cl)cc3)Cc2c1	-	MMV Malaria Box	-
MMV675939	FC(F)(F)C1=CC=C(NC2=CC(NC(C3=CC(C(F)(F)F)=CC=C3)=N4)=C4C=N2)N=C1	-	Literature[o]	-
MMV085071	COc1cncc(c1)-c1cncc(n1)N1CCN(CC1)c1ccncc1	-	MMV Pathogen Box	-
MMV668311	CNc1nc(NCCCN(C)C)c2sc(cc2n1)c3cccc(c3)C(F)(F)F	-	Literature[p]	-
MMV020746	Cc1ccc(Oc2ncccc2C(=O)Nc2cccc3cccnc23)c(C)c1	-	Literature[p]	TCMDC-125499
MMV667491	CN(C)CCCn1cnc2c(c1=N)C(c3ccc4ccccc4c3O2)c5ccc(cc5)OC	-	MMV Malaria Box	-
MMV006455	CCCN(CCC)CC(O)COC1=C(C=CC=C1)C(=O)NC1=CC=CC=C1	-	MMV Malaria Box	-
MMV022478	Clc1cccc(c1)-c1cnn2ccc(nc12)C(=O)Nc1ccc(cc1)N1CCNCC1.OC(C(F)(F)F)=O	-	MMV Pathogen Box	-
MMV007181	CC1=C2C=CC(O)=CC2=NC(NC2=CC=C(OCC3=CC=CC=C3)C=C2)=C1	-	MMV Malaria Box	-
MMV665971	CCOC(=O)C1=C(C)N=c2s\c(=C/c3cc(Cl)ccc3O)c(=O)n2C1c1ccc(OC)cc1	-	MMV Malaria Box	-
MMV665939	FC1=CC=C(C=C1)C(=O)NC1=C(SC=C1)C(=O)NC1CCCCC1	-	MMV Malaria Box	-
MMV019017	COCCNCC(O)CN1C2=CC=C(Cl)C=C2C2=C1C=CC(Cl)=C2	-	MMV Malaria Box	-
MMV000248	Cl.CCN(CC)CCn1c2ccccc2n(CC(O)c2ccc(Cl)c(Cl)c2)c1=N	-	MMV Malaria Box	-
MMV021735	CCCCCCCN(CC1=CC=C(OC(C)(C)C(=O)OCC)C=C1)C(=O)NC1=CC=C(Cl)C=C1OCC	-	Literature[p]	TCMDC-131919
MMV022224	[O-]C(=O)C(F)(F)F.CN(C)CC1=CC=C(C=C1)C1=CC2=C(N1)N=CC=C2C1=CC=C(CN(C)C)C=C1	-	Literature[p]	TCMDC-132409
MMV027496	COC1=C(OCCN(C)C)C=CC(=C1)C1=NC(=C(N1)C1=CC=CC=C1)C1=CC=CC=C1	-	Literature[p]	TCMDC-137716
MMV019555	Cl.C(CCCNc1c2CCCCc2nc2ccccc12)CCNc1c2CCCCc2nc2ccccc12	-	MMV Malaria Box	TCMDC-124183
MMV030666	CC(C)(C)OC(=O)N1CCN(CC1)C1=CC=CC=C1NC(=O)C1=C(OC2=CC=C(F)C=C2)C(=CC=C1)C(F)(F)F	-	Literature[p]	TCMDC-140951
MMV000787	CCCOCC1=C2C=CC=NC2=C(O)C(CN2CCN(CC2)C2=CC(Cl)=CC=C2)=C1	-	MMV Malaria Box	-

PI4K: Phosphatidylinositol-4-OH kinase, CPSF: Cleavage and polyadenylation specificity factor, ATP4: P-type cation translocating ATPase, DHODH: Dihydroorotate dehydrogenase, CYTB: Cytochrome B, DXR: 1-deoxy-D-xylulose-5-phosphate reductoisomerase; SMILES: simplified molecular line entry system.

[a]Slater, A.F., and Cerami, A. (1992). Inhibition by chloroquine of a novel haem polymerase enzyme activity in malaria trophozoites. Nature *355* , 167-169.

[b]Raynes, K., Foley, M., Tilley, L., and Deady, L.W. (1996). Novel bisquinoline antimalarials. Synthesis, antimalarial activity, and inhibition of haem polymerisation. Biochemical Pharmacology *52* , 551-559.

[c]Biot, C., Taramelli, D., Forfar-Bares, I., Maciejewski, L.A., Boyce, M., Nowogrocki, G., Brocard, J.S., Basilico, N., Olliaro, P., and Egan, T.J. (2005). Insights into the mechanism of action of ferroquine. Relationship between physicochemical properties and antiplasmodial activity. Molecular Pharmaceutics *2* , 185-193.

[d]Dubar, F., Egan, T.J., Pradines, B., Kuter, D., Ncokazi, K.K., Forge, D., Paul, J.F., Pierrot, C., Kalamou, H., Khalife, J., et al. (2011). The antimalarial ferroquine: role of the metal and intramolecular hydrogen bond in activity and resistance. ACS Chemical Biology *6* , 275-287.

[e]Atamna, H., Krugliak, M., Shalmiev, G., Deharo, E., Pescarmona, G., and Ginsburg, H. (1996). Mode of antimalarial effect of methylene blue and some of its analogues on Plasmodium falciparum in culture and their inhibition of P. vinckei petteri and P. yoelii nigeriensis in vivo. Biochemical Pharmacology *51* , 693-700.

[f]McNamara, C.W., Lee, M.C., Lim, C.S., Lim, S.H., Roland, J., Simon, O., Yeung, B.K., Chatterjee, A.K., McCormack, S.L., Manary, M.J., et al. (2013). Targeting Plasmodium PI(4)K to eliminate malaria. Nature *504* , 248-253.

[g]Sonoiki, E., Ng, C.L., Lee, M.C., Guo, D., Zhang, Y.K., Zhou, Y., Alley, M.R., Ahyong, V., Sanz, L.M., Lafuente-Monasterio, M.J., et al. (2017). A potent antimalarial benzoxaborole targets a Plasmodium falciparum cleavage and polyadenylation specificity factor homologue. Nature Communications *8* , 14574.

[h]Spillman, N.J., Allen, R.J., McNamara, C.W., Yeung, B.K., Winzeler, E.A., Diagana, T.T., and Kirk, K. (2013). Na(+) regulation in the malaria parasite Plasmodium falciparum involves the cation ATPase PfATP4 and is a target of the spiroindolone antimalarials. Cell Host Microbe *13* , 227-237.

[i]Stokes, B.H., Yoo, E., Murithi, J.M., Luth, M.R., Afanasyev, P., da Fonseca, P.C.A., Winzeler, E.A., Ng, C.L., Bogyo, M., and Fidock, D.A. (2019). Covalent Plasmodium falciparum-selective proteasome inhibitors exhibit a low propensity for generating resistance in vitro and synergize with multiple antimalarial agents. PLoS Pathog. 15, e1007722.

[j]Li, H., O'Donoghue, A.J., van der Linden, W.A., Xie, S.C., Yoo, E., Foe, I.T., Tilley, L., Craik, C.S., da Fonseca, P.C., and Bogyo, M. (2016). Structure- and function-based design of Plasmodium-selective proteasome inhibitors. Nature *530* , 233-236.

[k]Coteron, J.M., Marco, M., Esquivias, J., Deng, X., White, K.L., White, J., Koltun, M., El Mazouni, F., Kokkonda, S., Katneni, K., et al. (2011). Structure-guided lead optimization of triazolopyrimidine-ring substituents identifies potent Plasmodium falciparum dihydroorotate dehydrogenase inhibitors with clinical candidate potential. Journal of Medicinal Chemistry *54* , 5540-5561.

[l]Fry, M., and Pudney, M. (1992). Site of action of the antimalarial hydroxynaphthoquinone, 2-[trans-4-(4'-chlorophenyl) cyclohexyl]-3-hydroxy-1,4-naphthoquinone (566C80). Biochemical Pharmacology *43* , 1545-1553.

[m]Armstrong, C.M., Meyers, D.J., Imlay, L.S., Freel Meyers, C., and Odom, A.R. (2015). Resistance to the antimicrobial agent fosmidomycin and an FR900098 prodrug through mutations in the deoxyxylulose phosphate reductoisomerase gene (dxr). Antimicrobial Agents and Chemotherapy *59* , 5511-5519.

[n]Vanaerschot, M., Lucantoni, L., Li, T., Combrinck, J.M., Ruecker, A., Kumar, T.R.S., Rubiano, K., Ferreira, P.E., Siciliano, G., Gulati, S., et al. (2017). Hexahydroquinolines are antimalarial candidates with potent blood-stage and transmission-blocking activity. Nature Microbiology *2* , 1403-1414.

[o]Nchinda, A.T., Le Manach, C., Paquet, T., Gonzalez Cabrera, D., Wicht, K.J., Brunschwig C., Njoroge, M., Abay, E., Taylor, D., Lawrence, N., et al. (2018). Identification of fast-acting 2,6-disubstituted imidazopyridines that are efficacious in the in vivo humanized Plasmodium falciparum NODscidIL2Rγ^{null} mouse model of Malaria. Journal of Medicineal Chemistry *61* , 4213-4227.

[p]Corey, V.C., Lukens, A.K., Istvan, E.S., Lee, M.C.S., Franco, V., Magistrado, P., Coburn-Flynn, O., Sakata-Kato, T., Gnadig, N., et al. (2016). A broad analysis of resistance development in the malaria parasite. Nature Communications *15* , 11901.

Table S3 (related to Figure 4). Assessment of compound solubility by UV/vis spectroscopy.

Compound	Wavelength						Ratio fil./std.	Solubility
	280	300	320	340	360	800		
	Absorbance (AU)							
**DHA fil.**	2.351	0.462	0.250	0.179	0.124	0.041	1.02	≥ 500 μM
**DHA std.**	2.314	0.456	0.244	0.173	0.120	0.042		
**Chloroquine fil.**	2.637	1.170	2.155	2.075	0.203	0.042	0.91	≥ 100 μM and ≤ 500 μM
**Chloroquine std.**	2.747	1.331	2.562	2.178	0.204	0.041		
**Piperaquine fil.**	2.322	0.473	0.269	0.189	0.129	0.041	0.69	≥ 100 μM and ≤ 500 μM
**Piperaquine std.**	2.694	0.822	0.646	0.555	0.462	0.304		
**MMV007181 fil.**	2.346	0.470	0.259	0.194	0.139	0.042	0.69	≥ 100 μM and ≤ 500 μM
**MMV007181 std.**	2.677	0.754	0.534	0.551	0.508	0.131		
**MMV000442 fil.**	2.517	0.606	0.380	0.310	0.248	0.086	0.74	≥ 100 μM and ≤ 500 μM
**MMV000442 std.**	2.754	0.898	0.711	0.660	0.619	0.274		
**MMV006455 fil.**	2.810	0.904	0.336	0.186	0.130	0.041	1.00	≥ 500 μM
**MMV006455 std.**	2.826	0.928	0.329	0.175	0.120	0.041		

Fil.: filtered; std: standard.

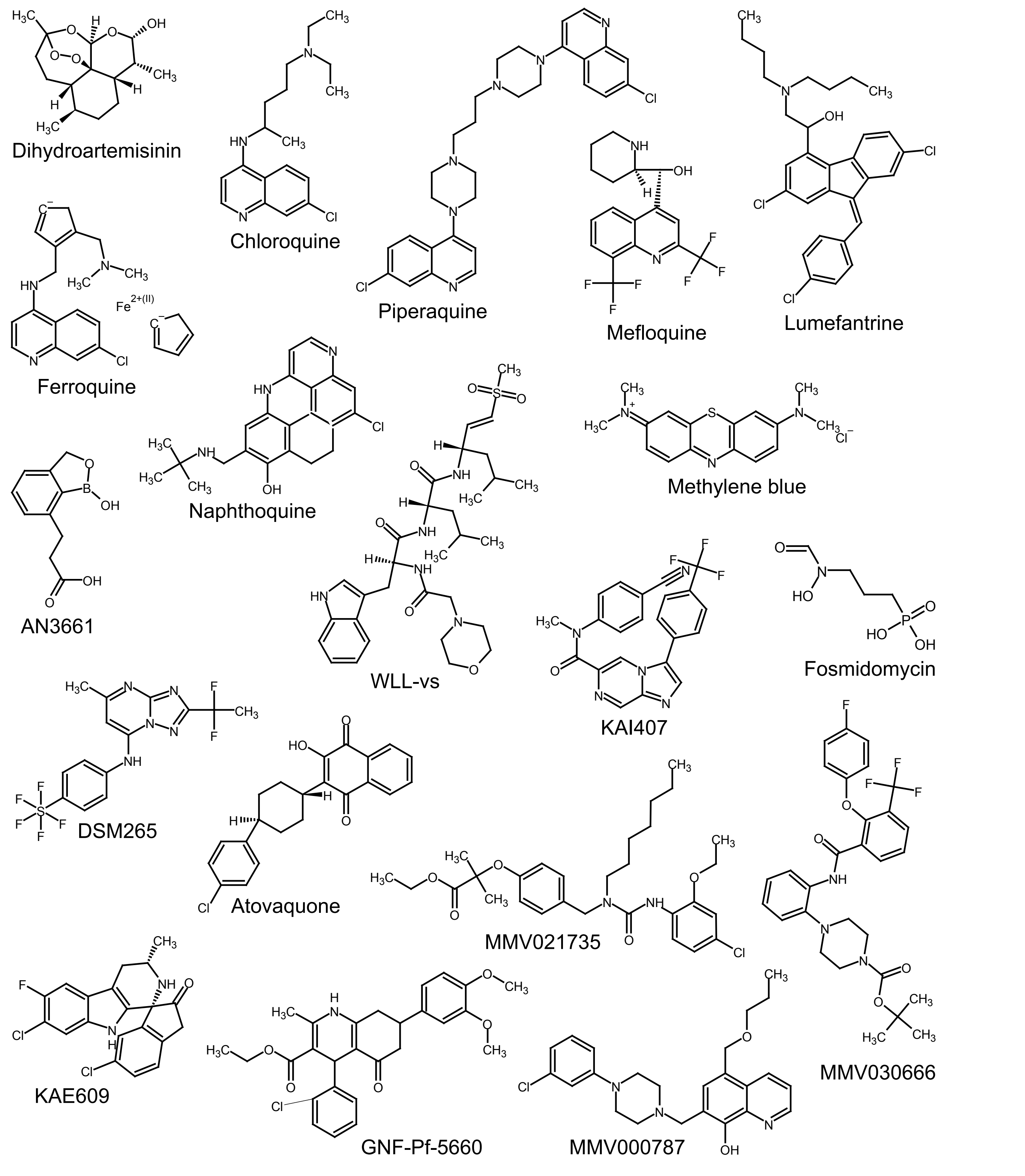

Figure S1 (related to Figures 1–6). Structures of the tested antimalarials, part 1. References on mode of action can be found in Table S2.

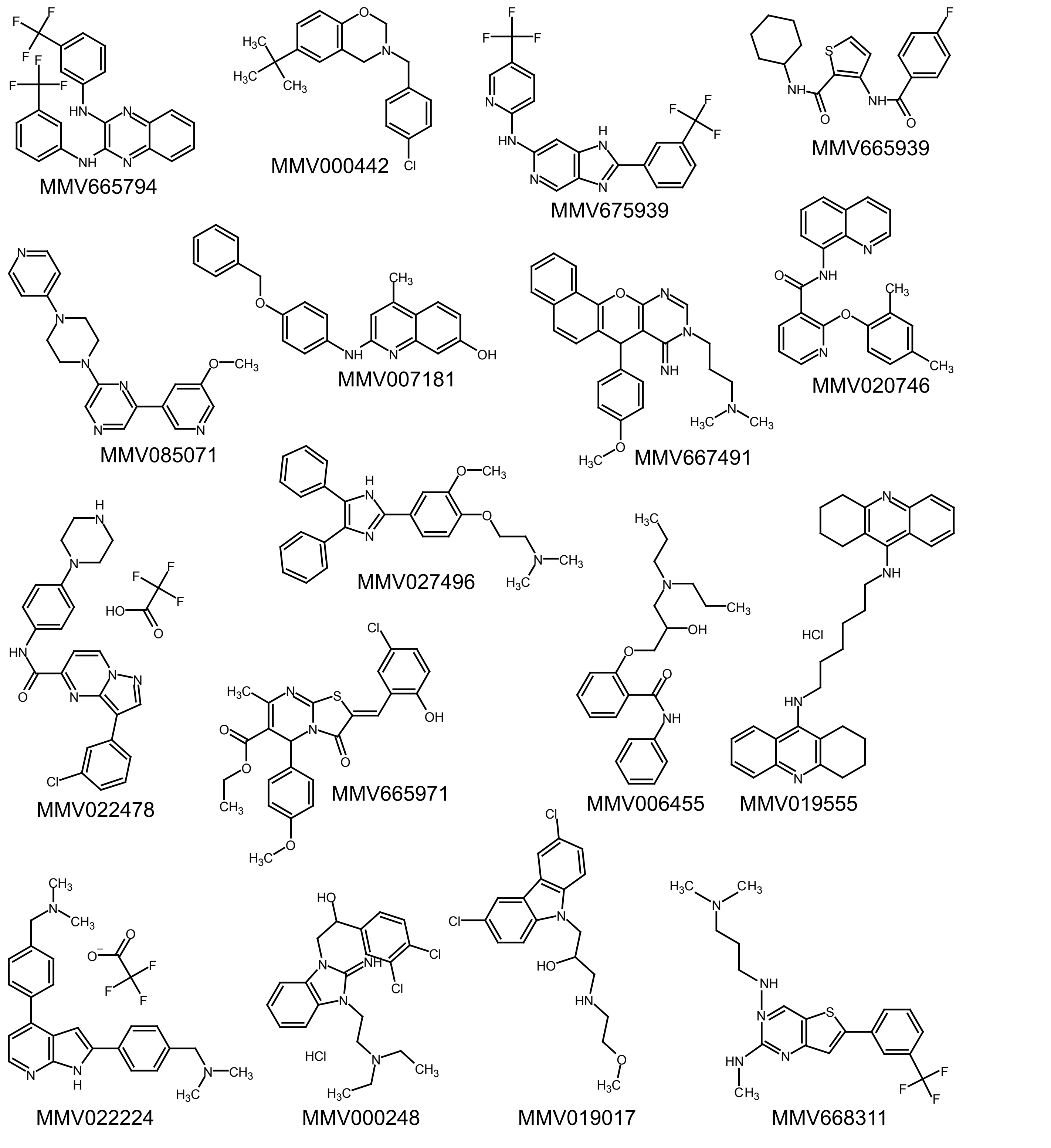

Figure S2 (related to Figures 1–6). Structures of the tested antimalarials, part 2. References on mode of action can be found in Table S2.

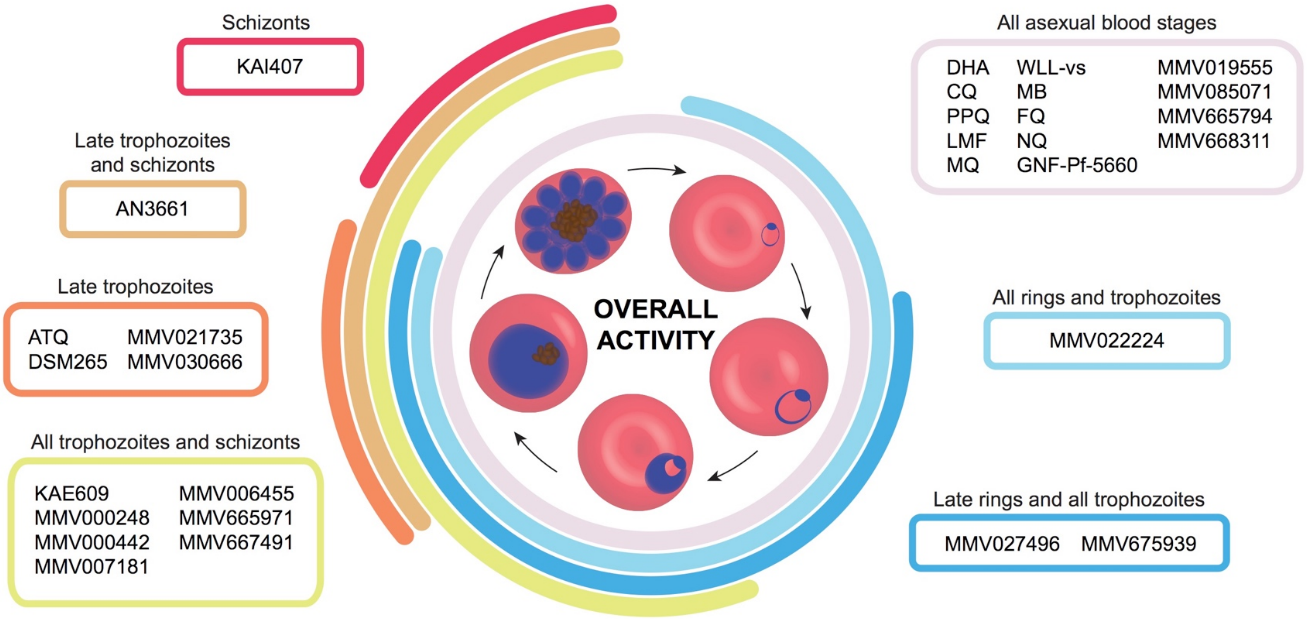

Figure S3 (related to Figure 6). Overall activity profile of compounds. Overall activity is defined as the stages with IC_{50}^{8h} values < 1 µM. Fosmidomycin, MMV000787, MMV019017, MMV020746, MMV022478 and MMV665939 are not depicted, as all stages showed IC_{50}^{8hr} values > 1 µM. MMV006455, MMV000442, MMV007181 and MMV665971 are omitted from panel A due to incomplete killing at individual stages. DHA: dihydroartemisinin; CQ: chloroquine; PPQ: piperaquine; LMF: lumefantrine; MQ: mefloquine; MB: methylene blue; FQ: ferroquine; NQ: naphthoquine; ATQ: atovaquone.

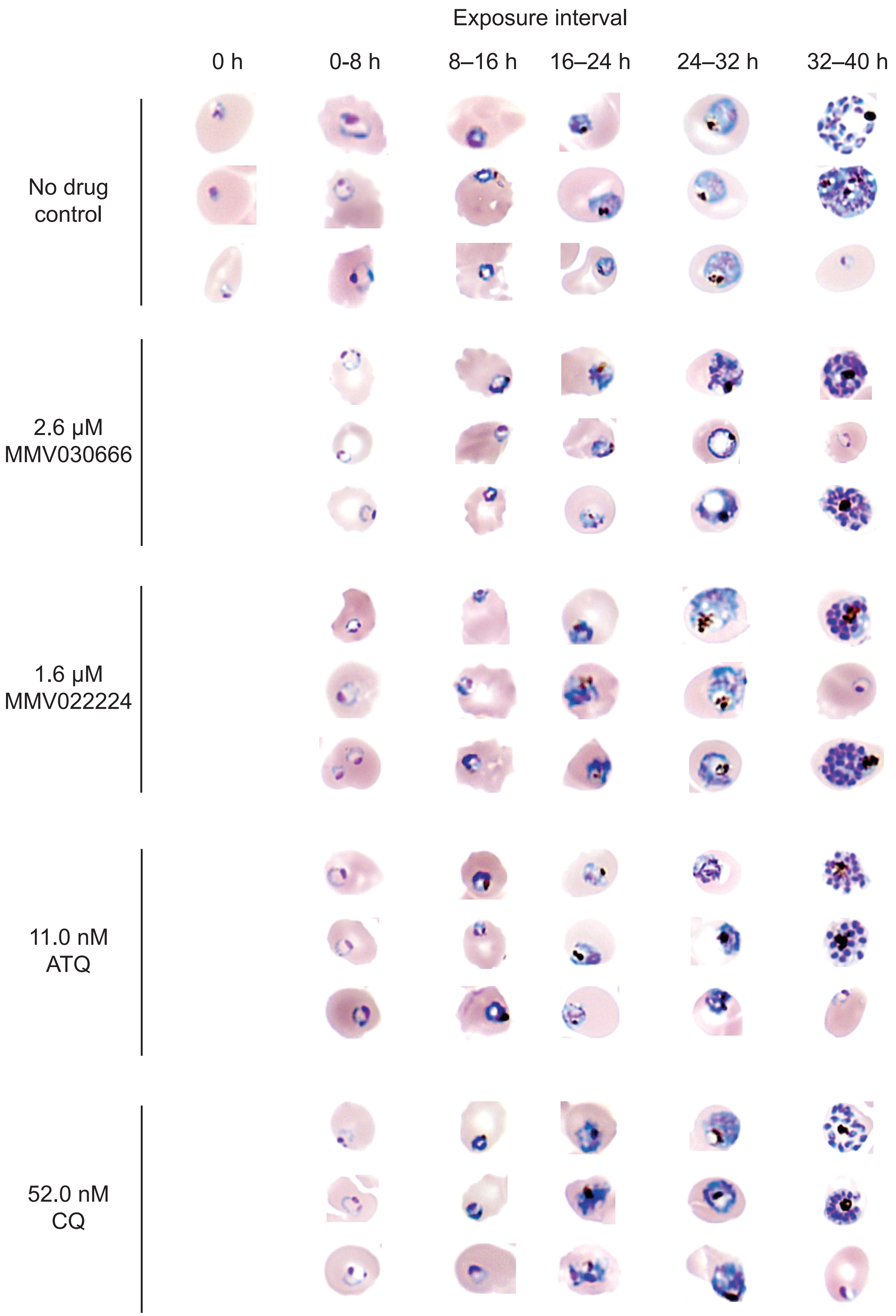

Figure S4 (related to Figure 2 and Figure 4). **Microscopical studies confirm the stage specificity profiles of MMV030666 and MMV022224, using ATQ and CQ as controls.** Synchronized parasites were exposed to 3× their lowest IC_{50}^{8h} at the indicated life stages, and were assessed at the end of each exposure. ATQ: atovaquone, CQ: chloroquine.
